# Novel acridine-based thiosemicarbazones as ‘turn-on' chemosensors for selective recognition of fluoride anion: a spectroscopic and theoretical study

**DOI:** 10.1098/rsos.180646

**Published:** 2018-07-04

**Authors:** Ibanga Okon Isaac, Iqra Munir, Mariya al-Rashida, Syed Abid Ali, Zahid Shafiq, Muhammad Islam, Ralf Ludwig, Khurshid Ayub, Khalid Mohammed Khan, Abdul Hameed

**Affiliations:** 1 H. E. J. Research Institute of Chemistry, International Center for Chemical and Biological Sciences, University of Karachi, Karachi 75270, Pakistan; 2 Department of Chemistry, COMSATS Institute of Information Technology, Abbottabad, Khyber Pakhtunkhwa 22060, Pakistan; 3 Department of Chemistry, Forman Christian College (A Chartered University), Ferozepur Road, Lahore, Pakistan; 4 Institute of Chemical Sciences, Organic Chemistry Division, Bahauddin Zakariya University, Multan 60800, Pakistan; 5 Leibniz-Institut für Katalyse e. V. an der Universität Rostock, Albert-Einstein-Strasse 29a, 18059 Rostock, Germany; 6 Department of Physical Chemistry, University of Rostock, Dr.-Lorenz-Weg 1, 18059 Rostock, Germany; 7 Department of Clinical Pharmacy, Institute for Research and Medical Consultations (IRMC), Imam Abdulrahman Bin Faisal University, PO Box 31441, Dammam, Saudi Arabia

**Keywords:** acridine, thiosemicarbazides, fluoride (F^−^), UV absorption, fluorescence, density functional theory calculations

## Abstract

New thiosemicarbazide-linked acridines **3a–c** were prepared and investigated as chemosensors for the detection of biologically and environmentally important anions. The compounds **3a–c** were found selective for fluoride (F^−^) with no affinity for other anions, i.e. ^−^OAc, Br^−^, I^−^, HSO_4_^−^, SO_4_^2−^, PO_4_^3−^, ClO_3_^−^, ClO_4_^−^, CN^−^ and SCN^−^. Further, upon the gradual addition of a fluoride anion (F^−^) source (tetrabutylammonium fluoride), a well-defined change in colour of the solution of probes **3a–c** was observed. The anion-sensing process was studied in detail via UV–visible absorption, fluorescence and ^1^H-NMR experiments. Moreover, during the synthesis of acridine probes **3a–c** nickel fluoride (NiF_2_), a rarely explored transition metal fluoride salt, was used as the catalyst. Theoretical studies via density functional theory were also carried out to further investigate the sensing and anion (F^−^) selectivity pattern of these probes.

## 1. Introduction

Acridine, a tricyclic nitrogen heterocyclic compound, has been frequently used as a dye for dyeing silks, wood and leather. In medicinal chemistry, many acridine derivatives have found applications as drugs such as acriflavine (a topical antiseptic) and quinacrine (an antiprotozoal drug). Most of the acridine derivatives and their metal complexes also act as DNA intercalating agents. However, the use of acridine-based compounds as chemosensors has not yet been explored for anionic analysis. With this interest, we aimed to synthesize acridine-based compounds functionalized with semicarbazide moieties. Previous studies have provided evidence regarding the role of semicarbazide moiety in anion sensing [1–4]. Chemosensing carries unique significance in monitoring biological processes, in clinical diagnostics and in monitoring environmental factors. Anion sensing via colorimetric assay has gained much popularity for qualitative and quantitative ion sensing for the purpose of regulating ion concentration in biological systems, water samples and clinical analysis [5–9]. Many different types of small molecules have been discovered as anion-sensing probes (or chemosensors) for the detection of biologically important anions such as halides, phosphates, acetates, ascorbate, citrates, etc. [4,10–12]. Fluoride carries unique importance among biologically important anions due to its applications in dental care products such as toothpaste, a material used in the daily routine in human life, and in treatment of osteoporosis [13–15].

**Figure 1.**
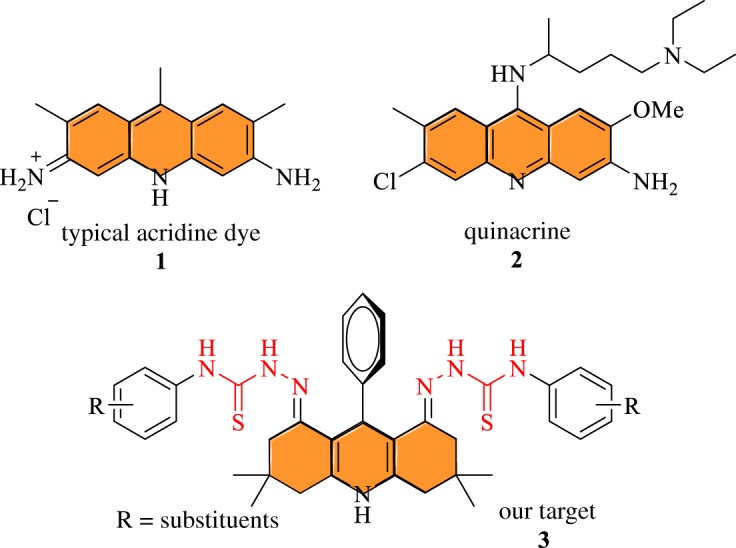
Acridine-based compounds.

Traditionally, the known techniques for ion detection are spectroscopy, chromatography and ion-selective electrodes which require expensive instrumentation and extensive and destructive sample preparation, and hence cannot be employed for cell or tissue imaging [16]. In recent times, the use of small molecules as fluorescent probes for ion detection in biological, environmental and industrial systems has rapidly gained in popularity [10,17–29]. Anion detection via small molecules holds enormous potential for biologically important anions, such as fluoride (F^−^), acetate (AcO^−^), sulfate, phosphate, pyrophosphate, ascorbate, citrate, etc. [10,30,31]. In this regard, molecules having biologically and environmentally friendly scaffolds are more desirable for anion-sensing process, because they offer least toxicity. In this study, we have functionalized the acridine scaffold with a thiosemicarbazide moiety, which acts as an ion-binding site during the anion-sensing process (figure 1).

## 2. Results and discussion

### 2.1. Chemistry

**Scheme 1.**
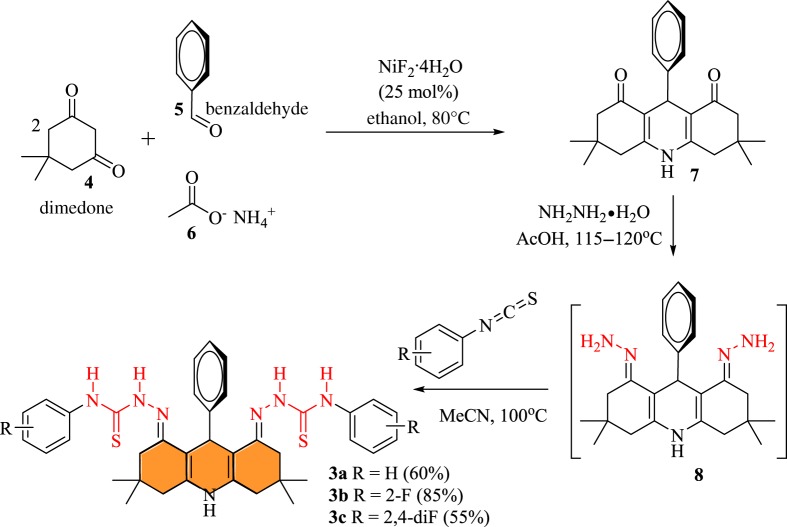
Synthetic layout for acridine-based probes **3a–c**.

The synthetic layout of targeted thiosemicarbazide-linked acridine is given in scheme 1. The core acridine template was constructed by reacting 2 mol of 5,5-dimethylcyclohexane-1,3-dione (dimedone), 1 mol of benzaldehyde and 1 mol of ammonium acetate in the presence of nickel fluoride tetrahydrate as a catalyst. It is important to note here that NiF_2_·4H_2_O is not well explored as a catalyst in organic synthesis, though such type of reactions have been explored with some traditional fluorides such as CsF or tetrabutylammonium fluoride (TBAF) [32–34]. The reaction proceeded smoothly in the presence of NiF_2_·4H_2_O (25 mol%) as the catalyst to furnish the acridine scaffold **7** with a simple work-up and washing with *n*-hexane. The use of transition metal fluoride (NiF_2_) as a new catalytic source to promote organic reaction could serve as a substitute for common fluoride salts, i.e. CsF, KF, etc. Further, in order to endow the acridine scaffold with a semicarbazide moiety, it was treated with excess of hydrazine hydrate (80%), and subsequently with phenyl isocyanate in acetonitrile at 80–85°C, until the disappearance of the starting materials, as monitored by thin-layer chromatography (TLC). The desired thiosemicarbazide-linked compounds, as anion-sensing probes **3a–c**, were obtained in a 55–85% yield (scheme 1).

In the ^1^H-NMR spectrum of acridine-based thiosemicarbazone **3b** (as a selected example), characteristic broad signals for two N*H* groups were observed at *δ*_H_ 10.32 and 9.23 ppm, while the broad signal for N*H* of the acridine scaffold appeared at *δ*_H_ 8.80 ppm. The peaks of all (13) aromatic protons appeared between *δ*_H_ 7.65 and 6.91 ppm. A sharp singlet for the H-9 proton appeared at *δ*_H_ 5.27 ppm. Further, the signals for four methylene groups (CH_2_)_4_ appeared as doublets at (i) 2.49 ppm (obscured by the dimethylsulfoxide (DMSO) signal), (ii) 2.31 ppm, (iii) 2.23 ppm and (iv) 2.16 ppm. Two singlets for each of two methyl groups at position C-3/6 appeared at *δ*_H_ 1.03 ppm and 0.82 ppm. The structure of **3b** was further confirmed with two-dimensional NMR techniques. The heteronuclear single quantum coherence spectrum showed direction correlation of the methine (CH-9) group at 35.6 ppm, while the heteronuclear multiple bond correlation (HMBC) spectrum showed correlations of H-9 with C-1/8, C-2′′/6′′, C-10/13 and C-11/12. The two N*H*_a_ protons showed correlations with C-1^′^, C-6^′^ carbons atoms, while the N*H*_b_ protons showed correlations with C-1/8 carbon atoms in the HMBC spectrum. The proton of acridine-N*H* has a correlation with C-10/13 carbon atoms. Further details of NMR signals and correlations have been provided in table 1. The infrared (IR) signals of **3b** have been provided in the experimental section. The mass of **3b** via electrospray ionization (ESI+) was found at *m/z* 684.1 (M + H).

**Table 1.** Characteristic ^1^H-NMR, ^13^C-NMR and HMBC correlation data of **3b**. 
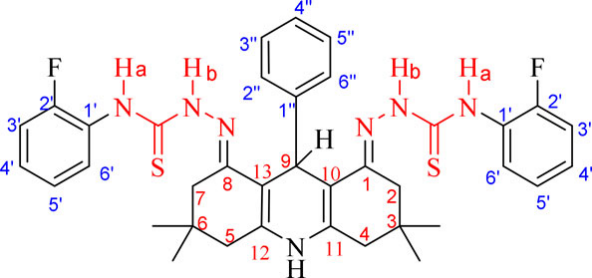
.

atom no.	*δ*_H_ (m, *J* in Hz)	*δ*_C_	HMBC correlations
1/8 (C)	—	152.1	—
2/7 (CH_2_)	2.49 (obscured by DMSO signal) /2.23 (d, *J* = 16.4)	38.2	NH_b_, C-1/8, C-4/5, C-3/6, C-10/13, (CH_3_)_2_
3/6 (C)	—	30.3	—
4/5 (CH_2_)	2.31 (d, *J* = 16.4)/2.16 (d, *J* = 16.4)	39.3	NH, C-2/7, C-3/6, C-10/13, C-11/12, (CH_3_)_2_
9 (CH)	5.27	35.9	C-1/8, C-2′′/6′′, C-10/13, C-11/12
10/13 (C)	—	106.7	—
11/12 (C)	—	140.1	—
1^′^/1^′^ (C)	—	126.6/126.5	—
2^′^/2^′^ (C)	—	157.2/154.2	—
3^′^/3^′^ (CH)	6.92 (m)	115.2/115.1	C-1^′^, C-2^′^, C-5^′^
4^′^/4^′^ (CH)	7.22–7.18 (m)	126.9/126.8	C-2^′^, C-6^′^
5^′^/5^′^ (CH)	7.10 (t, *J* = 7.6)	123.6	C-1^′^, C-3^′^
6^′^/6^′^ (CH)	7.64 (t, *J* = 6.8)	128.2	C-2^′^, C-4^′^
1′′ (C)	—	148.4	—
2′′/6′′ (CH)	7.31 (d, *J* = 7.2)	128.0	C-4′′, C6′′/C-2′′, C-4′′
3′′/4′′/5′′ (CH)	6.96–6.90 (m)	127.2/125.2	C-1′′, C-5′′/C-2′′, C-6′′ /C-1′′, C-3′′
N*H*_a_	9.23	—	C-1^′^, C-6^′^
N*H*_b_	10.32	—	C-1/8, C=S
N*H*	8.79	—	C-10/13, C-4/5
C=S	—	175.8	—
C(CH_3_)_2_	1.03	—	C-2/7, C-4/5
C(CH_3_)_2_	0.82	—	C-2/7, C-4/5

### 2.2. Anion sensing

**Figure 2.**
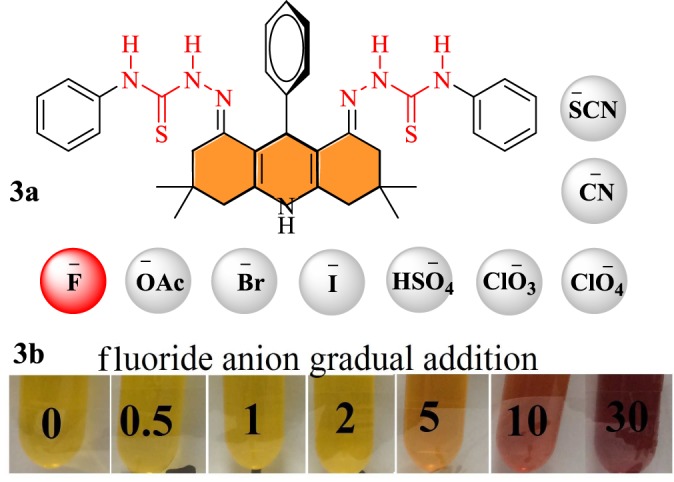
Acridine-based probe **3a**: selective sensing of F^−^ among different anions; **3b**: gradual addition of fluoride (0–30 equiv.) anions (naked eye view).

For colorimetric sensing, the pattern of receptors/chemosensors **3a–c** for different biologically important anions, i.e. F^−^, ^−^OAc, Br^−^, I^−^, HSO_4_^−^, ClO_3_^−^, CClO_4_^−^, CN^−^ and SCN^−^, has been studied via UV–visible (UV–Vis) spectroscopy. The anion-sensing study with thiosemicarbazide-linked acridines **3a–c** showed high selectivity for fluoride when compared with other anions such as acetate, bromide, iodide, bisulfate, chlorate, perchlorate, cyanide and thiocyanate. An increasing amount of different anions (up to 30 equivalents) was added to the solution of receptors **3a–c** (4 × 10^−5^ M). The change in colour of the receptor solution from yellow to reddish brown, to dark brown (up to 30 equivalents) was only observed upon the addition of fluoride (figure 2), whereas in the case of other anions, no change in colour was observed. The UV spectral profile of a model receptor **3a** showed absorption peaks at 320, 415 and 485 nm in DMSO diluted in acetonitrile solvent due to π–π* and *n*–π* transitions. A detailed record of UV–Vis absorption peaks for all receptors **3a–c** is given in electronic supplementary material, table S1.

**Figure 3.**
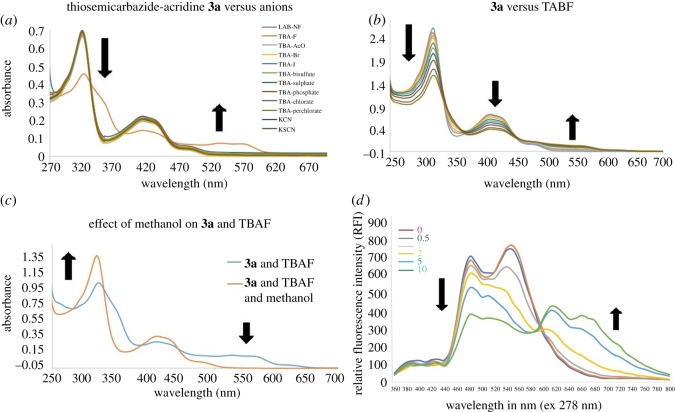
(*a*) UV absorption spectra of probe **3a**, (*b*) absorption spectra of probe **3a** upon gradual increase of fluoride addition, (*c*) upon dilution in methanol absorbance of **3a** is restored and (*d*) fluorescence spectra of receptor **3a** (excitation at 270 nm) upon the gradual increase of fluoride anions by using DMSO/acetonitrile as the solvent.

The titration of thiosemicarbazide–acridine receptor **3a** as a model sensor against different anions showed high selectivity for fluoride anion (red line) with a clear shift in the UV spectrum (figure 3*a*). Further the UV–Vis titration experiments of receptor **3a** upon gradual increase of fluoride anions (0–30 equiv.) showed a decrease in intensity at 320, 415 and 480 nm with a concomitant appearance of a new red-shifted band at 550 nm (figure 3*b*). The formation of three isobestic points at 332, 375 and 455 nm clearly indicates the formation of new species upon the addition of a fluoride anion source, TBAF. The UV–Vis spectra of receptor **3b** having mono-fluoride substituent on the phenyl ring were found to be almost similar in selectivity and detection pattern for fluoride anions as that of probe **3a**, when titrated with different anion solutions such as F^−^, ^−^OAc, Br^−^, I^−^, HSO_4_^−^, SO_4_^2−^, PO_4_^3−^, ClO_3_^−^, ClO_4_^−^, CN^−^, SCN^−^ and H_2_PO_4_^−^ (electronic supplementary material, figure S4). Moreover, the titration of di-fluoride-substituted receptor **3c** with different anions also showed it to be selective only for fluoride anion (electronic supplementary material, figure S5). Selectivity of all probes **3a–b** towards fluoride anion could be due to the small size and basic nature of fluoride that helps to remove acidic hydrogen atoms from the thiosemicarbazide moiety of receptor **3c**, resulting in observed changes in colour and UV–Vis spectrum (electronic supplementary material, figure S5).

Moreover, a continuous variation method, known as Job's plot, was used to determine the stoichiometric ratio between receptor **3a** and the fluoride anion during titration experiments. The Job's plot of probe **3a** and fluoride showed maxima at a mole fraction greater than 0.8 (electronic supplementary material, figure S2), while for **3b** and **3c** between 0.7 and 0.8 (electronic supplementary material, figure S3 and S5), which indicates the interaction/abstraction of more than one fluoride anion [3]. The binding constant calculated by using the Benesi–Hildebrand equation [35] was found to be 2.66 × 10^2^ M^−1^ with a limit of detection (LOD) of 6.879 × 10^−5^ M for probe **3a** (electronic supplementary material, figure S2). For probes **3a** and **3c**, the binding constant and LOD values are given in table 2.

**Table 2.** Binding constants and detection limits of receptors **3a–c** for fluoride anion detection.

comp. no.	binding constant (M^−1^)	detection limit (M)
**3a**	2.66 × 10^2^	6.879 × 10^−5^
**3b**	4.48 × 10^3^	9.08 × 10^−5^
**3c**	2.86 × 10^3^	6.17 × 10^−5^

Interestingly, it has been also observed that upon dilution of **3a** receptor–anion solution in methanol, the absorption intensity at 360 nm was restored with a concomitant disappearance of the band at 460 nm (figure 3*c*). These results suggested the degradation of the receptor–anion complex via re-protonation of NH groups in the presence of protic (i.e. MeOH) solvent. The other probes **3b** and **3c** also showed the same pattern of UV–Vis absorption spectra (electronic supplementary material, figures S4c and S5c). The sensitivity of probes **3a–c** was further explored via a fluorescence spectral study. The solutions of probes **3a** and **3b** were prepared followed by gradual addition of fluoride ions (0 to 10 equiv.), and were then subjected to fluorescence spectroscopic studies. The fluorescence spectra of probe **3b** upon excitation at 278 nm showed a gradual decease in the peak at 530 nm with a concomitant appearance of a new peak at 610 nm (electronic supplementary material, figure S4d). The pattern of probe **3a** was also observed in the same way, with a little difference in that it showed two peaks at 480 and 560 nm (figure 3*d*). Moreover, the applicability of the thiosemicarbazone-linked acridine, i.e. probe **3b,** has been explored with toothpaste samples (electronic supplementary material, figure S4e). The variations in UV spectra clearly indicate the fluoride-sensing ability of the synthesized linked acridine–thiosemicarbazone receptors.

**Figure 4.**
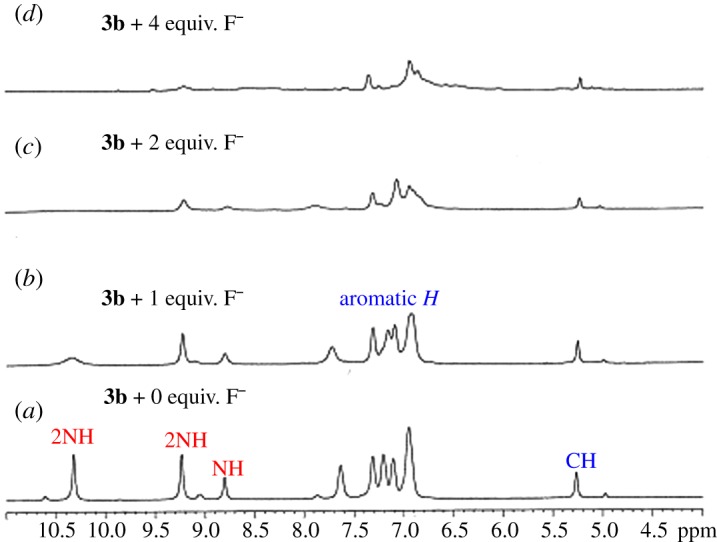
Fluoride (F^−^) anion gradual addition to probe **3b**: ^1^H-NMR spectra (*a*) without F^−^ anion, (*b*) 1 equiv., (*c*) 2 equiv. and (*d*) 4 equiv. of F^−^source (TBAF) in DMSO-*d_6_* as the solvent.

**Scheme 2.**
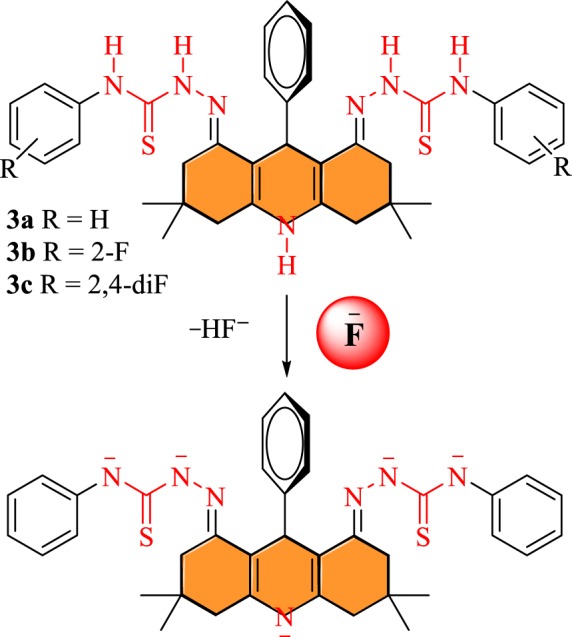
Fluoride interaction with thiosemicarbazone-linked acridine.

The interaction of semicarbazide-linked acridines **3a–c** with fluoride anions was further investigated by ^1^H-NMR study. The receptor **3b** was treated with a fluoride anion source (TBAF) in DMSO-*d*_6_. The fluoride source was added gradually to the NMR sample of receptor **3b** in DMSO-*d*_6_. ^1^H-NMR spectra were recorded before fluoride addition and then after adding different concentrations (0, 1, 2 and 4 equiv. of TABF) (figure 4). The area between *δ* 3 and 10 ppm in the ^1^H-NMR spectrum of receptor was zoomed to monitor the changes in N*H* signals of receptor **3b**. The spectrum of **3b** showed signals for N*H* groups at *δ* 10.32 (2N*H*), *δ* 9.23 (2N*H*) and *δ* 8.79 (1N*H*) ppm before addition of fluoride anion (TBAF). Upon addition of 1–2 equiv. of fluoride anions to the solution of receptor **3b,** a drastic decrease in the intensity of signals for N*H* protons (figure 4*b*) was observed. The signals for N*H* protons completely disappeared after addition of 4 equiv. of fluoride ions to receptor **3b** (figure 4*c*). The disappearance of characteristic signal for N*H* protons of model receptor **3b** upon the gradual addition of fluoride anion strongly suggested the abstraction of more than one N*H* proton by fluoride anions (scheme 2).

The interaction between a model probe **3a** and fluoride anions was also studied by ^1^H-NMR spectroscopy (electronic supplementary material, figure S1). The amount of TBAF as the fluoride anion source was gradually increased (0‒4 equiv.) to observe the changes in ^1^H-NMR signals. The signals for all N*H* groups at *δ* 10.17, *δ* 9.48 and *δ* 8.79 ppm for probe **3a** were significantly deceased and completely disappeared after the addition of 4 equiv. of fluoride anions (TBAF). The abstraction of N*H* protons of **3b** occurred due to the strong basic effect of F^−^ with respect to other anions studied. The signal of C*H*-9 proton at *δ* 5.26 ppm shifted slightly up-field, which was considered insignificant. The sequential ^1^H-NMR data showed clearly the selective interaction of the fluoride anion with NH protons due to its strong basic characteristic compared to other anions, i.e. ^−^OAc, Br^−^, I^−^, HSO_4_^−^, SO_4_^2−^, PO_4_^3−^, ClO_3_^−^, ClO_4_^−^, CN^−^ and SCN^−^. Moreover, the presence of five NH groups in thiosemicarbazide-linked acridine as receptor **3a** bestows the ability to detect more than one fluoride anion during the sensing process.

### 2.3. Computational study

All calculations were performed with a Gaussian 09 suite of programs [36]. Geometries of thiosemicarbazide acridines and their complexes with various anions are optimized without any symmetry constraints by the Coulomb-attenuated method of B3LYP (CAM-B3LYP) at the 6–311G(d,p) basis set [37,38]. Frequency analysis has been performed to confirm these optimized geometries as true minima (no imaginary frequency). The interaction energies of thiosemicarbazide acridines with various anions are calculated by the following equation:

$$E_{\mathrm{int}}=E_{\mathrm{complex}}-(E_{\mathrm{tsc}}+E_{\mathrm{anion}}),$$

where *E*_complex_ is the energy of complex formed between thiosemicarbazide–acridine and anions, whereas *E*_tsc_ and *E*_anion_ are the energies of isolated thiosemicarbazide–acridine and anions, respectively.

The excitation energies were also calculated with time-dependent density functional theory (DFT) at the (TD)CAM-B3LYP/6–311G(d,p) [37,38] level of theory. The electronic properties were computed with the Coulomb-attenuated method (CAM), a hybrid functional that implements long-range correction of B3LYP for calculation of excitation energies. The CAM-B3LYP method was chosen to study the excited state because of the well-established accuracy of this method for a variety of classes of organic compounds [39–42]. The solvent effect was studied through the polarization continuum model. All calculations were performed in DMSO solvent. A total of 20 states are calculated with 50 : 50 singlet : triplet states.

**Figure 5.**
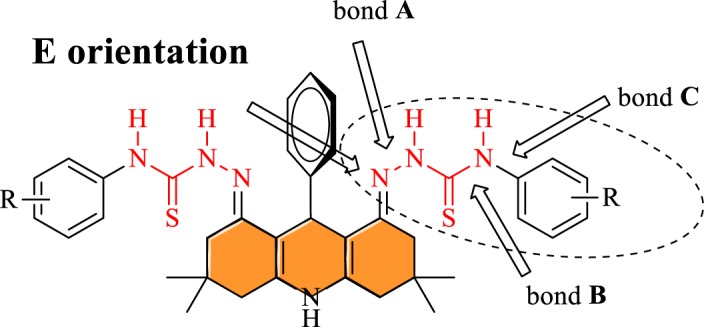
Description of degree of freedom for conformational and configurational isomerism.

**Figure 6.**
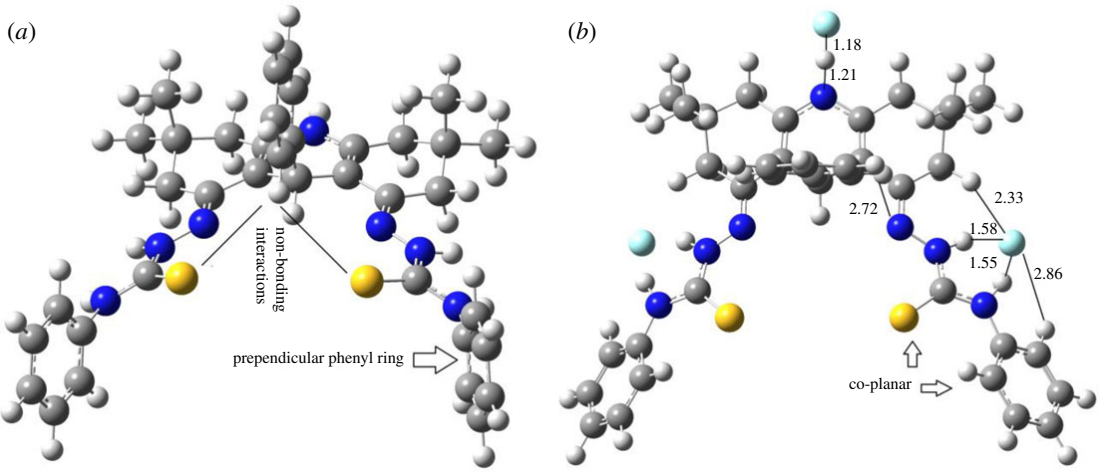
The optimized geometries of the lowest energy conformer of **3a** (*a*) and its 1 : 3 complex with fluoride ions (*b*).

DFT calculations have been performed to rationalize the selectivity of thiosemicarbazide–acridine-based probes for fluoride ions. In this regard, the calculations have been performed for complexation energies (of anions with the probe molecules) and UV–Vis absorption spectroscopic properties. In the first step, the most stable geometry of the probe molecule **3a** is searched. We have previously shown [43] that acridines prefer to adopt *E-*orientation of the N-group (thiosemicarbazide in our case, figure 5) even when the central ring bears hydrogen atoms. In our case, a phenyl ring is present in the central six-membered ring, which further limits the possibility of *Z*-orientation. Therefore, we started with the *E*-orientation of the thiosemicarbazide groups. The thiosemicarbazide group has several single bonds around which conformational scans produce a number of conformers. However, the bonds ‘A' and ‘C' are more important around which conformational scan has been performed to locate the low-energy conformers. The conformation of the lowest energy conformer (obtained through a conformational scan) is shown in figure 6, where the thio group interacts with the hydrogen atoms of the central phenyl ring. Moreover, the terminal phenyl ring is almost perpendicular to the plane of thiosemicarbazide (figure 6). The central C–N bonds (bond B) of the thiocarbazide adopt planar orientation because of the delocalization of electrons between nitrogen and the thio group.

In the next step, the interactions of various anions with the probe molecule are studied. The experimental results show that the probe molecule-to-anion ratio is more than three. Therefore, we started with the optimization of the 1 : 3 complex of probe **3a** with various anions. Analysis of the probe molecule reveals that there are three positions where anions can interact. These positions include N–H of the central ring, and H–N–C(S)–N–H of both thiosemicarbazide groups. An anion can interact with both N–H group of the H–N–C(S)–N–H moiety simultaneously to form a stable six-membered ring. The computational study is performed for complexes of probes **3a–c** with F^−^, ^−^OAc, Br^−^, Cl^−^ and CN^−^ anions, whereas other less basic and bulky anions (I^−^, HSO_4_^−^, ClO_3_^−^, ClO_4_^−^) are not considered. The optimized geometry of the 1 : 3 complex of probe (**3a**): F^−^ is shown in figure 6*b*.

Compound **3a** shows considerable changes in the geometry after complexation with fluoride ions. Important structural reorganizations during complexation are the changes in the orientations of the central and terminal phenyl rings. The fluoride ion interacts not only with the N–H protons of the thiosemicarbazide but also with the *ortho* hydrogen atoms of the terminal phenyl ring (figure 6*b*). Owing to this interaction, the terminal phenyl rings become coplanar with the thiosemicarbazide moiety, which causes the thio group to form loose non-bonding interactions with the central phenyl ring. As a result, the central phenyl ring gets rotated to have favourable interaction through its *ortho* hydrogen atoms with the nitrogen atom (of thiosemicarbazide) at a distance of 2.72 Å (figure 6*b*). The fluoride ion has strong proton affinity; therefore, the N–H bond of the central ring is considerably broken. The N–H and F–H bond lengths are 1.21 Å and 1.18 Å, respectively. The weakening of N–H bond (of the central ring) causes the nitrogen atom to become more electron-rich. The increased electron density causes changes in the electronic and spectroscopic properties of the complex (*vide infra*). The strong proton abstraction ability of F^−^ can be attributed to the high charge density on fluoride ions. The fluoride ion has −1 charge which is completely localized on it, whereas the negative charge on other ions (such as ^−^OAc, Br^−^, Cl^−^) is either more delocalized or diffused. The diffused or delocalized negative charges on other anions result in relatively weak interaction with the probe molecule (*vide infra* for binding energy calculations). These anions (^−^OAc, Br^−^, Cl^−^ and CN^−^) interact only with the N–H of the thiosemicarbazide without any involvement of the *ortho* hydrogen of the terminal phenyl ring. Therefore, the coplanarity of the terminal phenyl ring with the thiosemicarbazide moiety is not seen for these anions.

The binding energies are also calculated for these complexes, which reflect that the probe **3a** has the strongest binding affinity for the fluoride ion. The binding of three fluoride ions with a molecule of probe **3a** is exothermic by 383.97 kcal mol^−1^, which corresponds to an exothermicity of −127.99 kcal mol^−1^ per fluoride ion (table 3). The binding energy of the fluoride ion with probe **3a** is much stronger than that of other anions. The interaction energy of the fluoride ion with the acridine–thiosemicarbazide is comparable to a covalent bond. The higher binding energy of the fluoride ion with the probe molecule is consistent with the experimental observation where maximum response in sensing is seen for the fluoride ion. The binding energies of other anions range from −72 to −87 kcal mol^−1^ per anion, which is about 40–55 kcal mol^−1^ lower than that for the fluoride ion. Among other anions, the highest binding energy is calculated for acetate ion which may be attributed to the high electronegativity of oxygen combined with two binding sites (two oxygen atoms) per anion. Nitrile anion has a binding energy of −80.82 kcal mol^−1^ per anion, which amounts to −242.45 kcal mol^−1^ for three CN^−^ anions. The least binding energy is calculated for the interaction of bromide with the probe molecule, which may be attributed to the low charge density of bromide due to its large size.

**Table 3.** Total binding energies and binding energies per anion of probes **3a**–**c** for F^−^, Cl^−^, Br^−^, OAc^−^ and CN^−^.

	F^−^	Cl^−^	Br^−^	OAc^−^	CN^−^
total binding energies (kcal mol^−1^)					
**3a**	−383.97	−226.80	−216.86	−259.54	−242.45
**3b**	−383.25	−227.25	−217.25	−256.63	−242.14
**3c**	−386.43	−227.50	−217.49	−259.80	−242.24
binding energies per anion (kcal mol^−1^)					
**3a**	−127.99	−75.60	−72.29	−86.51	−80.82
**3b**	−127.75	−75.75	−72.42	−85.54	−80.72
**3c**	−128.81	−75.83	−72.50	−86.60	−80.75

A similar trend in the binding energies of different anions is observed for other probes **3b** and **3c**. The binding energies of the fluoride ion with probe **3b** and **3c** are −383.25 and −386.43 kcal mol^−1^, respectively, which amount to −127.75 and −128.81 kcal mol^−1^ per fluoride ion. The probe **3c** has highest binding energy for the fluoride ion when compared with **3b** and **3a**, which may be attributed to a greater electron-withdrawing effect of fluoride ions. Probe **3c** has relatively higher binding affinity for other anions compared to probe **3b** and **3a** but the difference is almost negligible. In general, the electron-withdrawing substituent on the terminal aromatic ring increases the binding affinities for different anions compared to the unsubstituted probe **3a**.

**Figure 7.**
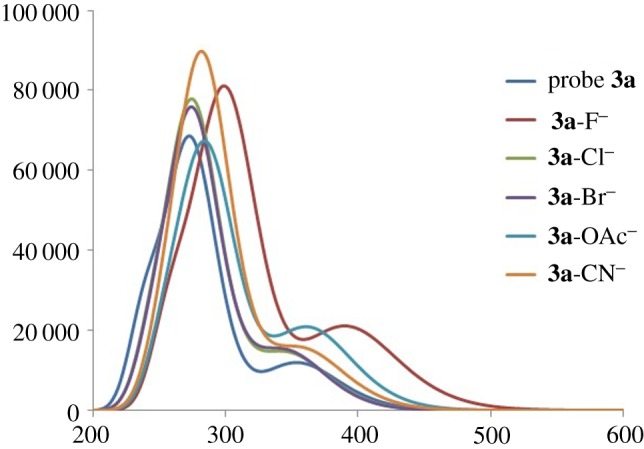
UV–Vis spectra of probe **3a** and its complexes with various anions, calculated at (TD)CAM-B3LYP/6-311G(d,p).

Finally, the UV–Vis spectra are calculated for *probe–anion* complexes, and they are compared to the UV–Vis spectrum of a pure probe molecule. The probe **3a** has two absorption bands appearing at 355.0 nm and 274.75 nm. The band at 355 nm has a tail which is stretched to 480 nm. The calculated UV–Vis spectrum at CAM-B3LYP is consistent with the experimental UV–Vis spectrum. Complexation of all anions except F^−^ does not cause any shift in the UV–Vis spectrum of the probe (figure 7).

For some anions such as ^−^OAc or CN^−^, the intensities of some of the absorption peaks are affected; however, the absorption maximum remains almost unaffected. The most significant change in the UV–Vis spectrum is observed for fluoride complexes where the UV–Vis spectrum is red-shifted, which is consistent with the experimental observations. The red-shifting of the UV–Vis spectrum of the **3a–F**^−^ complex is due to two factors: (i) the abstraction of proton by the fluoride ion leaves the nitrogen with more electron density which is now delocalized on the entire skeleton and (ii) the complexation of fluoride with the probe causes the planarization of the skeleton, particularly at the terminal phenyl ring (through interaction with *ortho* hydrogen atoms of the phenyl ring, *vide supra*). The other probes **3b** and **3c** also show similar selectivity for fluoride ions compared to other anions (table 4).

**Table 4.** Changes in the absorption maxima of **3a–c** on complexation with various anions, calculated at CAM-B3LYP/6–311G(d,p).

	${\lambda_{\max}}^{1}$	${\lambda_{\max}}^{2}$	${\lambda_{\max}}^{1}$	${\lambda_{\max}}^{2}$	${\lambda_{\max}}^{1}$	${\lambda_{\max}}^{2}$
*X*	**3a-X^−^** (1 : 3 complexes) *λ*_max_ (**3a**) = 355 and 274.75 nm		**3b-X^−^** (1 : 3 complexes) *λ*_max_ (**3b**) = 356.43 and 274.52 nm		**3c-X^−^** (1 : 3 complexes) *λ*_max_ (**3c**) = 356.38 and 274.27 nm	
F^−^	391.78	303.32	382.96	295.46	400.27	296.59
Cl^−^	347.95	276.75	345.36	272.94	345.76	272.82
Br^−^	346.37	276.76	344.64	272.90	345.93	272.92
OAc^−^	363.26	287.14	347.80	284.22	359.12	283.79
CN^−^	358.64	284.24	357.28	282.35	354.15	277.72

## 3. Conclusion

In summary, thiosemicarbazide-linked acridines **3a**–**c** have been synthesized and explored as chemosensors for biologically important anions, i.e. F^−^, ^−^OAc, Br^−^, I^−^, HSO_4_^−^, SO_4_^2−^, PO_4_^3−^, ClO_3_^−^, ClO_4_^−^, CN^−^ and SCN^−^. The core acridine was furnished from corresponding precursors, i.e. 5,5-dimethylcyclohexane-1,3-dione and ammonium acetate in the presence of nickel fluoride as the catalyst. The anion-sensing study was demonstrated via naked eye observation, UV–Vis absorption, fluorescence and ^1^H-NMR spectroscopic experiments. In the anion-sensing study, the receptors **3a–c** were found to be highly selective for the fluoride anion. This is possibly due to its small size and high basicity, which makes possible its strong interaction/abstraction of proton from receptors to induce changes in colour and signals/peaks in UV–Vis, fluorescence and ^1^H-NMR spectra. Upon the gradual addition of TBAF to acridine-based receptors, systemic changes in the UV–Vis and fluorescence spectra have been observed which illustrated the formation of new species during the sensing process. Moreover, the fluoride interaction with new thiosemcarbazide-linked acridines **3a–c** was further exploited via DFT.

## 4. Materials and methods (chemistry)

All the starting materials including 5,5-dimethyl-1,3-cyclohexanedione (95%), benzaldehyde, ammonium acetate, nickel fluoride (NiF_2_·4H_2_O) (99%), hydrazine hydrated (80%) and phenyl isothiocyanate (98%) were purchased from Sigma Aldrich or Merck and used directly for carrying out our reaction unless otherwise stated. Acetonitrile (HPLC grade), DMSO (HPLC grade), methanol (HPLC grade) and distilled or Milli-Q water were used. TLC was carried out with silica gel 60 aluminium-backed plates 0.063–0.200 mm (Merck, Germany). Analytical grade solvents such as ethyl acetate, diethyl ether, hexane and methanol were used as eluents for purification purpose through column chromatography. Round-bottomed flasks (5, 10, 25, 50, 100, 250 ml) were used. TLC visualization was carried out using UV lamp radiation at 254 nm. In addition, different spot test mixtures, such as basic potassium permanganate or vanillin, were also used. IR spectra were recorded with a Bruker Vector-22 spectrometer. The ^1^H-NMR spectra were recorded using Bruker Avance spectrometers at 300, 400, 500 and 600 MHz, while ^13^C-NMR spectra were recorded at 75, 100, 125 and 150 MHz in the appropriate deuterated solvent. The chemical shifts were recorded on the *δ*-scale (ppm) using residual solvents as an internal standard (DMSO: ^1^H 2.50, ^13^C 39.43; and CHCl_3_: ^1^H 7.26, ^13^C 77.16). Coupling constants were calculated in hertz (Hz) and multiplicities were labelled as s (singlet), d (doublet), t (triplet), q (quartet) and quint (quintet), and the prefixes br (broad) or app (apparent) were used. Mass spectra (EI^+^ and FAB) were recorded with a Finnigan MAT-321A, Germany. Melting points of solids were determined using a Stuart™ melting point SMP3 apparatus and are uncorrected.

### 4.1. General procedure for the synthesis of acridin-based thiosemicarbazones (**3a**–**c**)

To a 25 ml round-bottomed flask, equipped with a magnetic stirrer, dimedone (1.40 g, 10 mmol), ammonium acetate (0.57 g, 7.5 mmol), benzaldehyde (0.50 ml, 5 mmol), nickel(II) fluoride tetrahydrate (25 mol%) as the catalyst and ethanol (3 ml) were added. The reaction mixture was heated on an oil bath at 80°C with continuous stirring for 80 min until the complete consumption of the starting material, as monitored using TLC analysis. The resulting mixture was dissolved in methanol, and poured onto crushed ice with stirring to obtain precipitates of the product. The precipitates were washed with *n*-hexane to get rid of unreacted benzaldehyde. Pure corresponding acridine **7** was obtained in 86% yield (1.50 g).

The compound **7** (0.35 g, 1 mmol) was treated with hydrazine hydrate (10 ml) and acetic acid (2 ml) at 120°C for 28 h. Progress of the reaction was monitored by TLC analysis using 30% EtOAc in *n*-hexane. On completion of the reaction, water was added to get the precipitate of compound **8**, which was filtered and dried under vacuum. The yield was 97% (0.36 g) and the colour was golden yellow.

The synthesis of compounds **3a–c** was carried out by reacting intermediate **8** (1.0 equiv.) and the appropriate substituted aryl isocyanates or isothiocyanates (2 equiv.), which were taken in 15 ml acetonitrile and heated at 98–100°C for 13 h. The progress of the reaction was monitored through TLC analysis using a 0.20 mm thick precoated silica plate, and spots were visualized through UV light. On completion of the reaction, the mixtures were cooled down to ambient temperature, resulting in the formation of precipitates. The precipitates were filtered, washed with warm acetonitrile and dried to obtain the pure compounds **3a–c** in a 55–85% yield (scheme 1).

*2,2^′^-((1E,8E)-3,3,6,6-Tetramethyl-9-phenyl-3,4,6,7,9,10-hexahydroacridine-1,8(2H,5H)-diylidene)bis(N-phenylhydrazine-1-carbothioamide)* (**3a**). Light yellow solid, yield 60% (over three steps, 192 mg), MP 281–284°C. IR (*ν*_max_, cm^−1^): (KBr disc) 3420, 3272, 3216, 3093, 2956, 2925, 2863, 1645, 1596, 1526, 1479, 1405, 1367, 1316, 1260, 1240, 1193, 1156, 1074, 1001, 755, 697, 661. ^1^H-NMR (400 MHz, DMSO-*d_6_*): *δ*_H_ 10.17 (2H, brs, 2N*H*), 9.48 (2H, brs, 2N*H*), 8.79 (1H, brs, N*H*), 7.46–7.38 (6H, m, Ar*H*), 7.23–7.12 (6H, m, Ar*H*), 6.98–6.91 (3H, m, Ar*H*), 5.35 (1H, s, C*H*), 2.39 (obscured by DMSO signal, H-2/7), 2.32 (2H, d, *J* = 16.4 Hz, *H*-4/5), 2.24 (2H, d, *J* = 16.4 Hz, H-2/7), 2.16 (2H, d, *J* = 16.4 Hz, *H*-4/5), 1.04 (6H, s, (CH_3_)_2_), 0.81 (6H, s, (CH_3_)_2_). C^13^-NMR (100 MHz, DMSO-*d_6_*): *δ*_C_ 193.7 (C), 175.0 (C), 151.9 (C), 148.7 (C), 139.9 (C), 137.8 (C), 128.2 (CH), 128.0 (CH), 127.3 (CH), 125.3 (CH), 124.8 (CH), 124.5 (CH), 123.7 (CH), 106.7 (C), 39.6 (CH_2_ × 2), 38.3 (CH_2_ × 2), 35.9 (CH), 30.3 (C), 29.4 (CH_3_ × 2), 26.4 (CH_3_ × 2). ESI-MS: *m/z* 648.1 (M + H).

*2,2^′^-((1E,8E)-3,3,6,6-Tetramethyl-9-phenyl-3,4,6,7,9,10-hexahydroacridine-1,8(2H,5H)-diylidene)bis(N-(2-fluorophenyl)hydrazine-1-carbothioamide)* (**3b**). Yellow solid, yield 85% (over three steps), MP 275–278°C. IR (*ν*_max_, cm^−1^): (KBr disc) 3749, 3446, 3271, 3222, 3093, 2955, 2926, 1646, 1620, 1527, 1478, 1404, 1367, 1239, 1223, 1157, 1077, 1029, 811, 752, 709, 658. ^1^H-NMR (400 MHz, DMSO-*d_6_*): *δ*_H_ 10.32 (2H, brs, 2N*H*), 9.23 (2H, brs, 2N*H*), 8.80 (1H, brs, N*H*), 7.64 (2H, t, *J* = 6.8 Hz, Ar*H*), 7.31 (2H, d, *J* = 7.2 Hz, Ar*H*), 7.22–7.18 (2H, m, Ar*H*), 7.10 (2H, t, *J* = 7.6 Hz, Ar*H*), 6.96–6.91 (5H, m, Ar*H*), 5.27 (1H, s, C*H*), 2.49 (obscured by DMSO signal, H-2/7), 2.31 (2H, d, *J* = 16.4 Hz, *H*-4/5), 2.23 (2H, d, *J* = 16.4 Hz, H-2/7), 2.16 (2H, d, *J* = 16.4 Hz, *H*-4/5), 1.03 (6H, s, (CH_3_)_2_), 0.82 (6H, s, (CH_3_)_2_). C^13^-NMR (100 MHz, DMSO-*d_6_*): *δ*_C_ 193.7 (C), 175.8 (C), 157.2/154.8 (C), 152.1 (C), 148.4 (C), 140.1 (C), 128.2 (CH), 128.0 (CH), 127.2 (CH), 126.9/126.8 (CH), 126.6/126.5 (CH), 125.2 (CH), 123.6 (CH), 115.3/115.1 (CH), 39.6 (CH_2_ × 2), 29.2 (CH_2_ × 2), 35.9 (CH), 30.3 (C), 29.2 (CH_3_ × 2), 26.5 (CH_3_ × 2). ESI-MS: *m/z* 684.1 (M + H).

*2,2^′^-((1E,8E)-3,3,6,6-Tetramethyl-9-phenyl-3,4,6,7,9,10-hexahydroacridine-1,8(2H,5H)-diylidene)bis(N-(2,4-difluorophenyl)hydrazine-1-carbothioamide)* (**3c**). Yellow solid, yield 55% (over three steps), MP 279–281°C. IR (*ν*_max_, cm^−1^): (KBr disc) 3440, 3273, 2957, 1644, 1612, 1529, 1478, 1308, 1237, 1146, 1086, 965, 848, 765, 710. ^1^H-NMR (400 MHz, DMSO-*d_6_*): *δ*_H_ 10.21 (2H, brs, 2N*H*), 9.39 (2H, brs, 2N*H*), 8.68 (1H, brs, N*H*), 7.34–7.29 (4H, m, Ar*H*), 7.04–6.98 (4H, m, Ar*H*), 6.94 (3H, m, Ar*H*), 5.42 (1H, s, C*H*), 2.31–2.06 (8H, m, (CH_2_)_4_), 1.01 (6H, s, (CH_3_)_2_), 0.83 (6H, s, (CH_3_)_2_). ESI-MS: *m/z* 720.1 (M + H).

## Supplementary Material

Revised-Acridine-Thiosemicarbazones _NMR spectra and other

## Data accessibility

Our supplementary data including absorption/fluorescence spectral data and NMR spectra of compounds **3a–c** are deposited at Dryad (http://dx.doi.org/10.5061/dryad.9nq2kc4).
