# Supplementary material for: Novel acridine-based thiosemicarbazones as ‘turn-on' chemosensors for selective recognition of fluoride anion: a spectroscopic and theoretical study
Source: R Soc Open Sci. 2018 Jul 4;5(7):180646. doi: 10.1098/rsos.180646 (PMC6083729; doi:10.1098/rsos.180646)
Supplement: Revised-Acridine-Thiosemicarbazones _NMR spectra and other [file rsos180646supp1.docx]

**Novel Acridine-Based Thiosemicarbazones as “Turn-on” Chemosensors for Selective Recognition of Fluoride anion: A Spectroscopic and Theoretical Study**

Ibanga Okon Isaac,^a^ Iqra Munir,^a^ Mariya al-Rashida,^c^ Syed Abid Ali,^a^ Zahid Shafiq,^d^ Muhammad Islam,^d^ Ralf Ludwig,^e,f^ Khurshid Ayub,^b^* Khalid Mohammed Khan,^a,g^ and Abdul Hameed**^[[1]](#footnote-1)^***^a^

^a^H. E. J. Research Institute of Chemistry, International Center for Chemical and Biological Sciences, University of Karachi, Karachi-75270, Pakistan

^b^Department of Chemistry, COMSATS Institute of Information Technology, Abbottabad, KPK, Pakistan 22060

^c^Department of Chemistry, Forman Christian College (A Chartered University), Ferozepur Road, Lahore, Pakistan

^d^Institute of Chemical Sciences, Organic Chemistry Division, Bahauddin Zakariya University, Multan 60800, Pakistan

^e^Leibniz-Institut für Katalyse e. V. an der Universität Rostock, Albert-Einstein-Str. 29a, 18059 Rostock, Germany

^f^Department of Physical Chemistry, University of Rostock, Dr.-Lorenz-Weg 1, 18059 Rostock, Germany

^g^Department of Clinical Pharmacy, Institute for Research and Medical Consultations (IRMC), Imam Abdulrahman Bin Faisal University, P.O. Box 31441, Dammam, Saudi Arabia


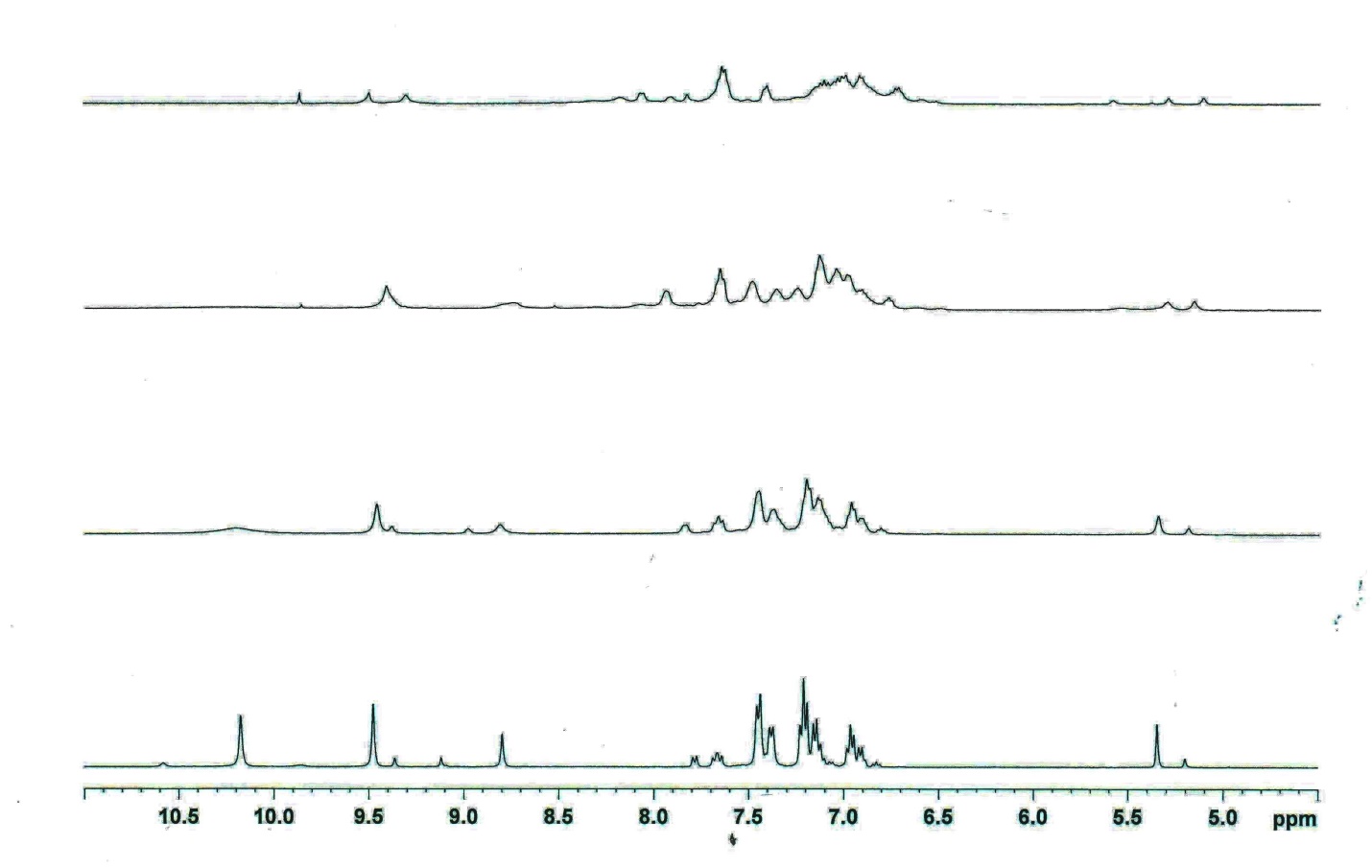


**3a** + 1 equiv. Fˉ

**3a** + 2 equiv. Fˉ

**3a** + 4 equiv. Fˉ

**3a** + 0 equiv. Fˉ

**Figure-S1**: Fluoride (Fˉ) anion gradual addition to probe **3a**: ^1^H NMR spectra; a) without Fˉ anion and b) 1 equiv. c) 2 equiv. d) 4 equiv. of F ˉsource (TBAF) in DMSO- *d_6_* as solvent.

**Table-S1**: UV-Vis absorption/fluorescence bands for probes **1-3** in DMSO/acetonitrile as solvent.

| **Probes** | **Absorptions Bands**  **(nm)** | | **Fluorescence**  **(Ex: 278 nm)** |
| --- | --- | --- | --- |
| **3a** | 320 | 415, 485 | 570, 670 |
| **3b** | 320 | 410 | 480, 570 |
| **3b** | 320 | 410 | - |

**Job Plot of 3a**





**LOD calculations of 3a**

**
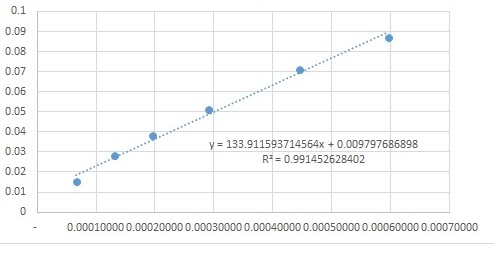
**

**Figure-S2:** Job’s plot and LOD of receptors **3a**.

**Job Plot of 3b**





**LOD calculations of 3b**

**
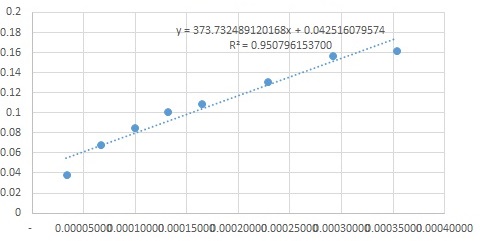
**

**Figure-S3:** Job’s plot and LOD of receptors **3b**.


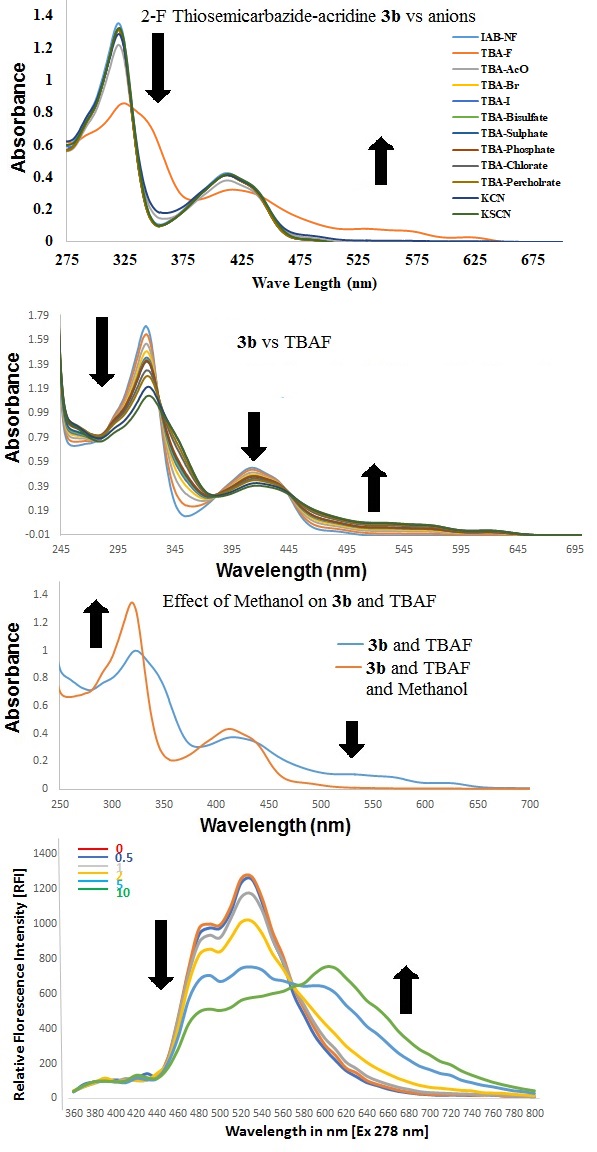


d)

b)

a)

c)

e)

Prob **3b**

Prob **3b** and

Toothpaste sample

Prob **3b** with toothpaste sample

**
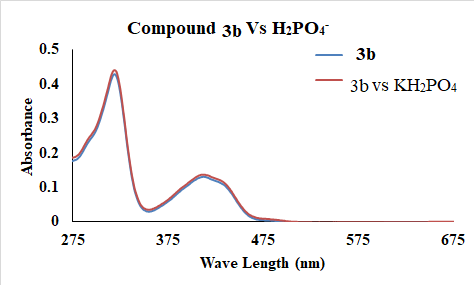
**

f)

**Figure-S4:** a) UV-Absorption spectra of probes **3b;** b) Absorption spectra of probe **3b** upon gradual increase of fluoride addition; c) Upon dilution in methanol absorbance restored **3b**; d) Fluorescence spectra of receptor **3b** (excitation at 270 nm) upon the gradual increase of fluoride anion by using DMSO/acetonitrile as solvent; e) UV-Absorption spectra of probes **3b** with toothpaste sample; f) Prob **3b** vs potassium dihydrogen phosphate (30 eq) in acetonitrile: water (9:1).

a)

b)

c)

**

**

d)

e)

**Figure-S5:** a) Fluoride detection by Probes **3c** a) UV-Absorption spectra of probes **3c** b) Absorption spectra of probe **3c** upon gradual increase of fluoride addition c) Upon dilution in methanol absorbance restored **3c**; d) Job’s plot and e) LOD of receptors **3b**.


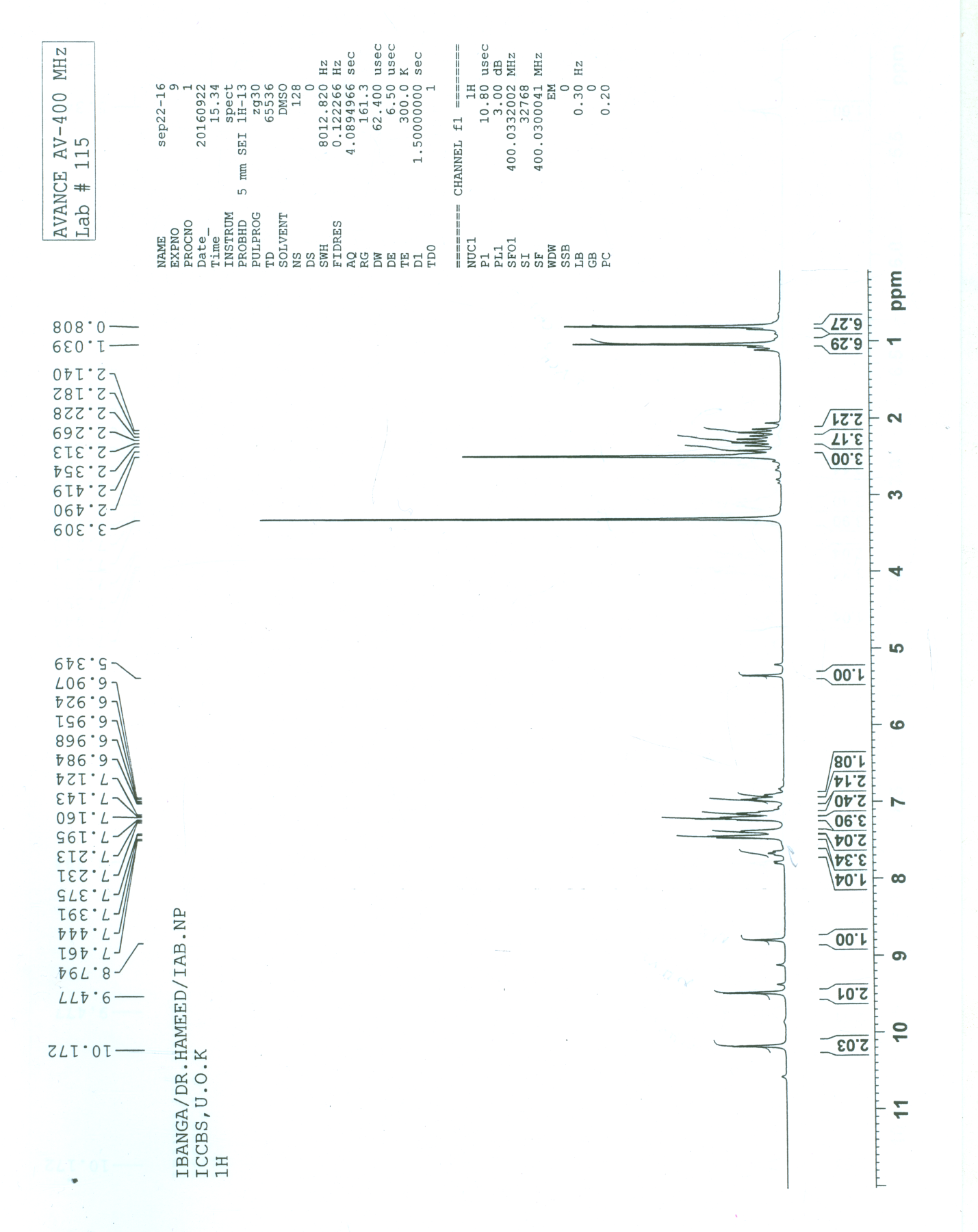

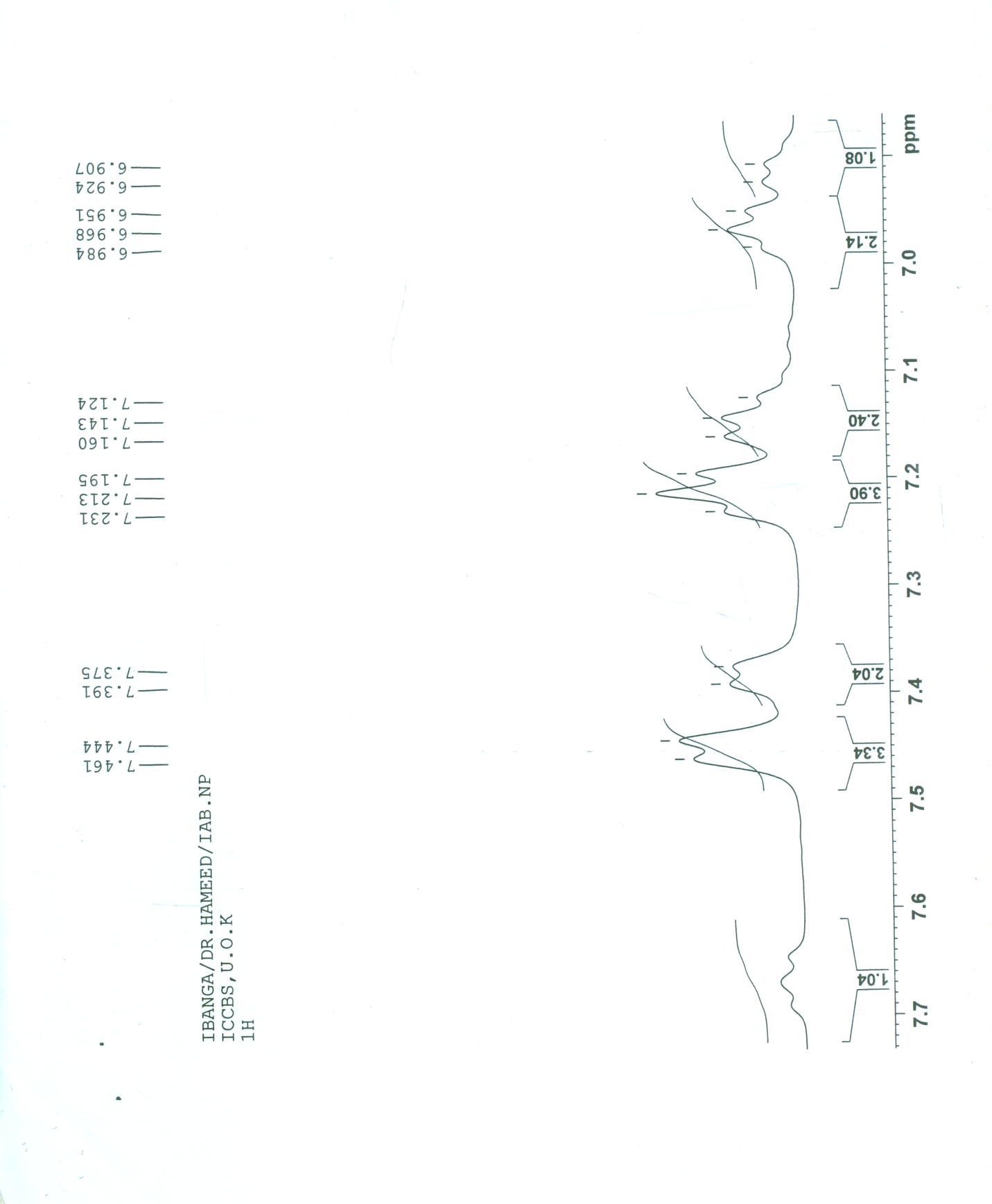

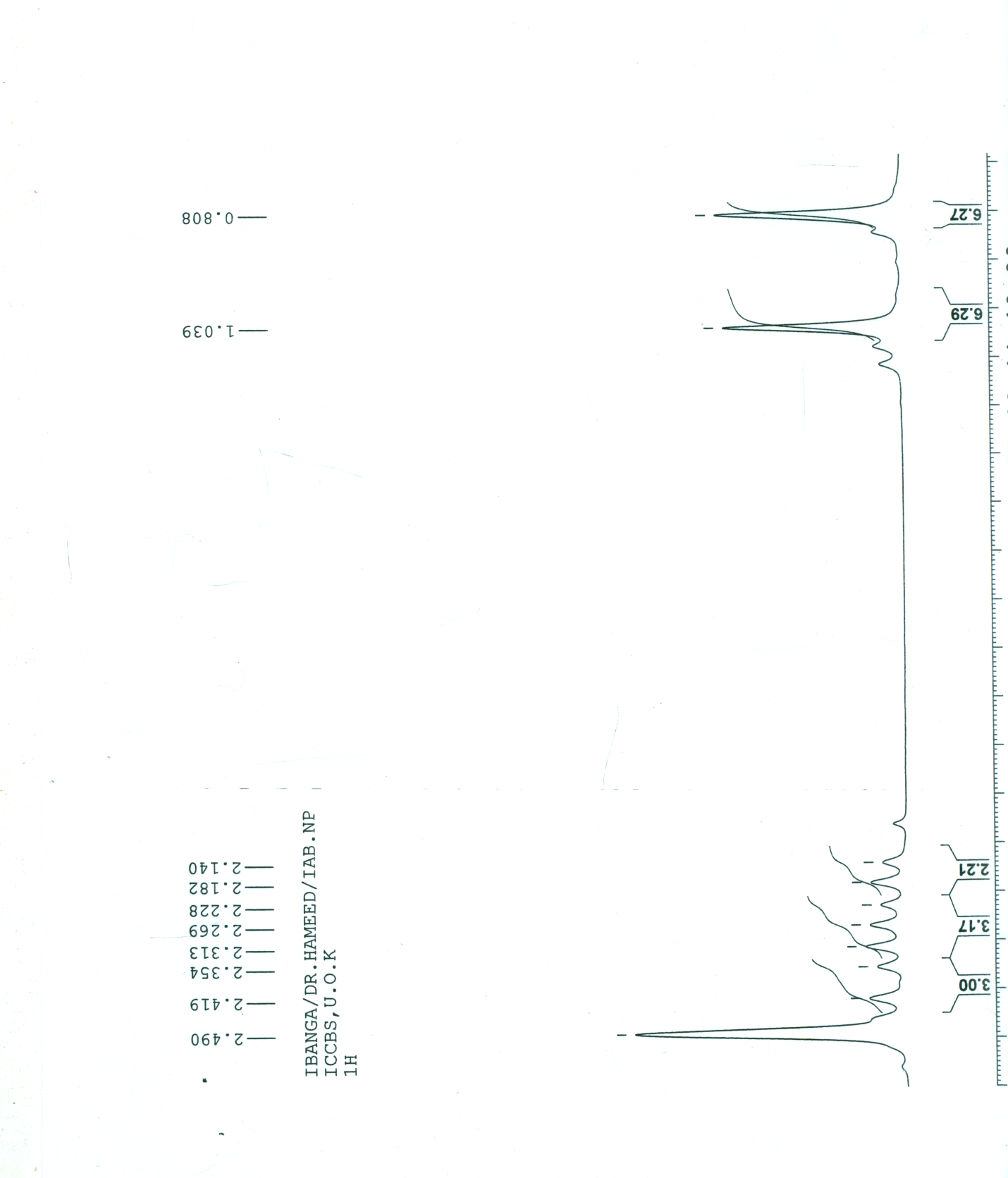

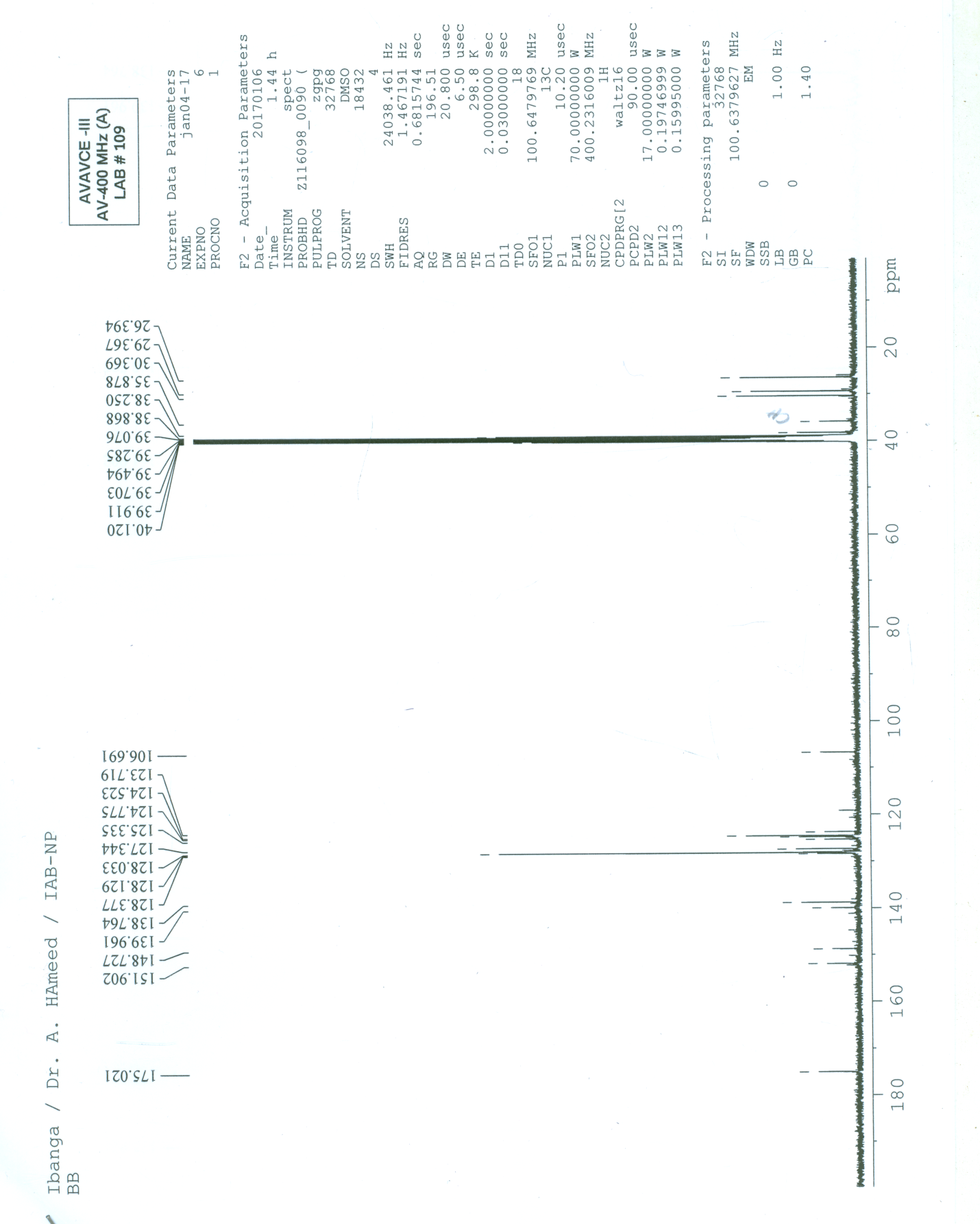

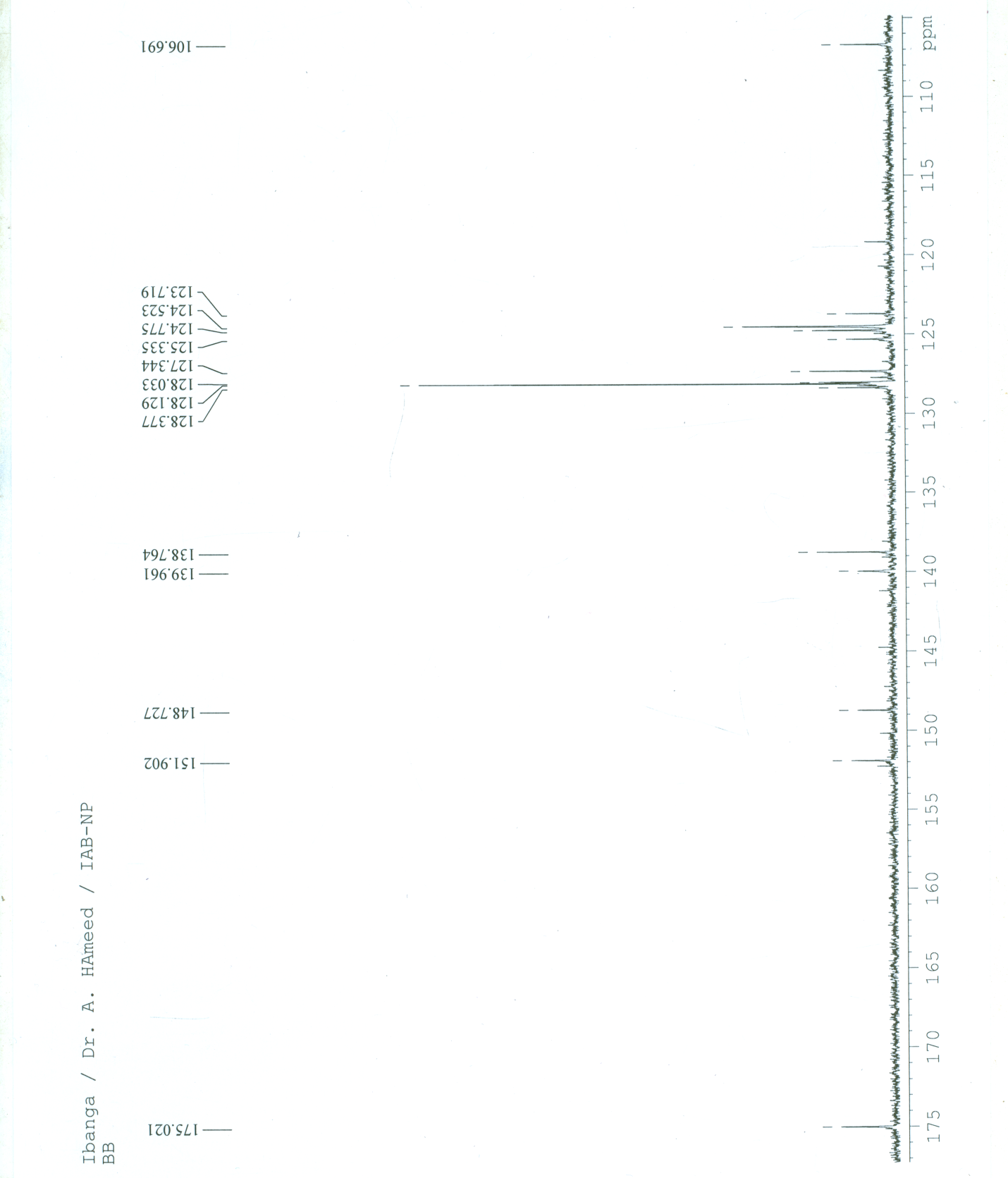

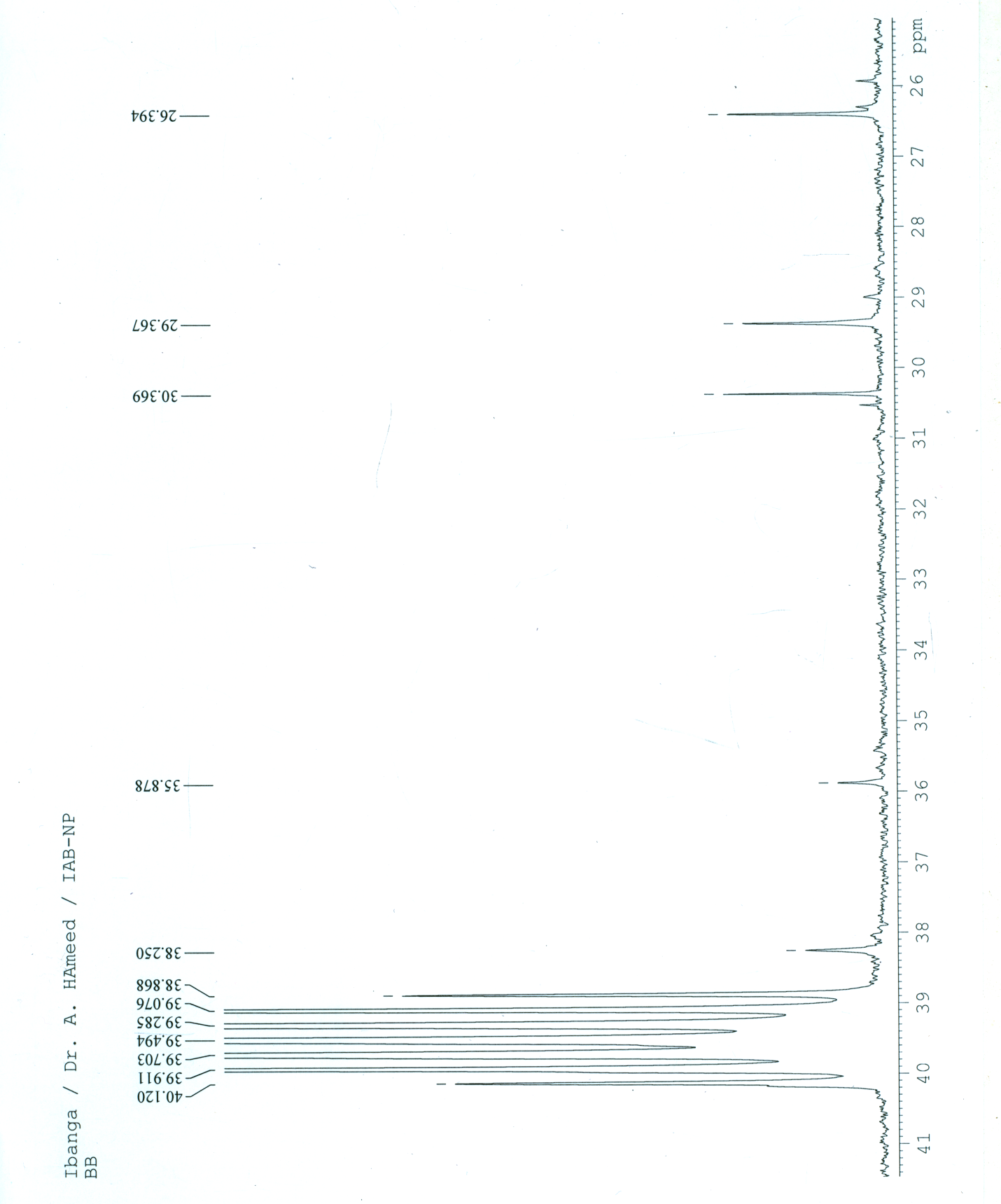


**
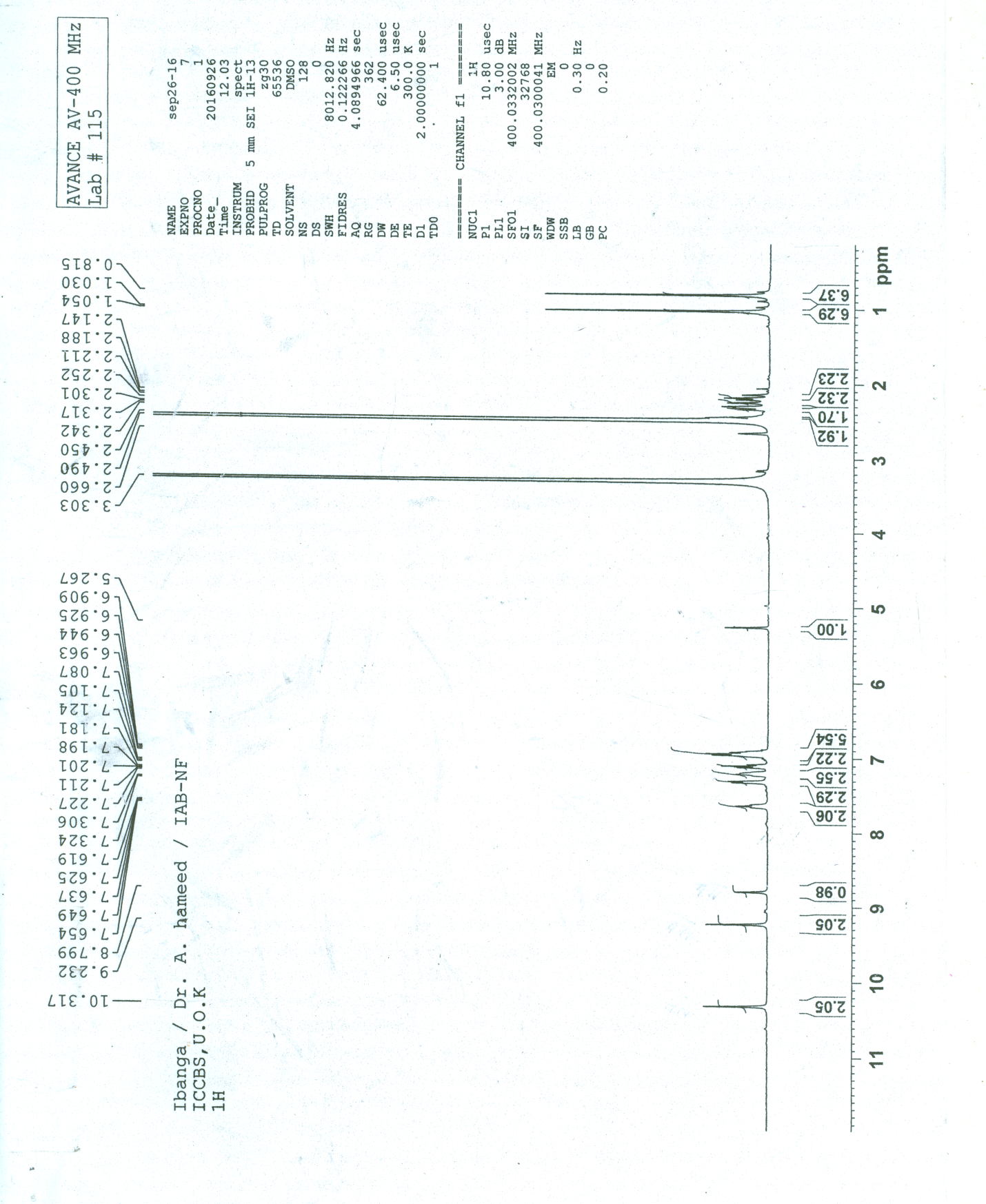

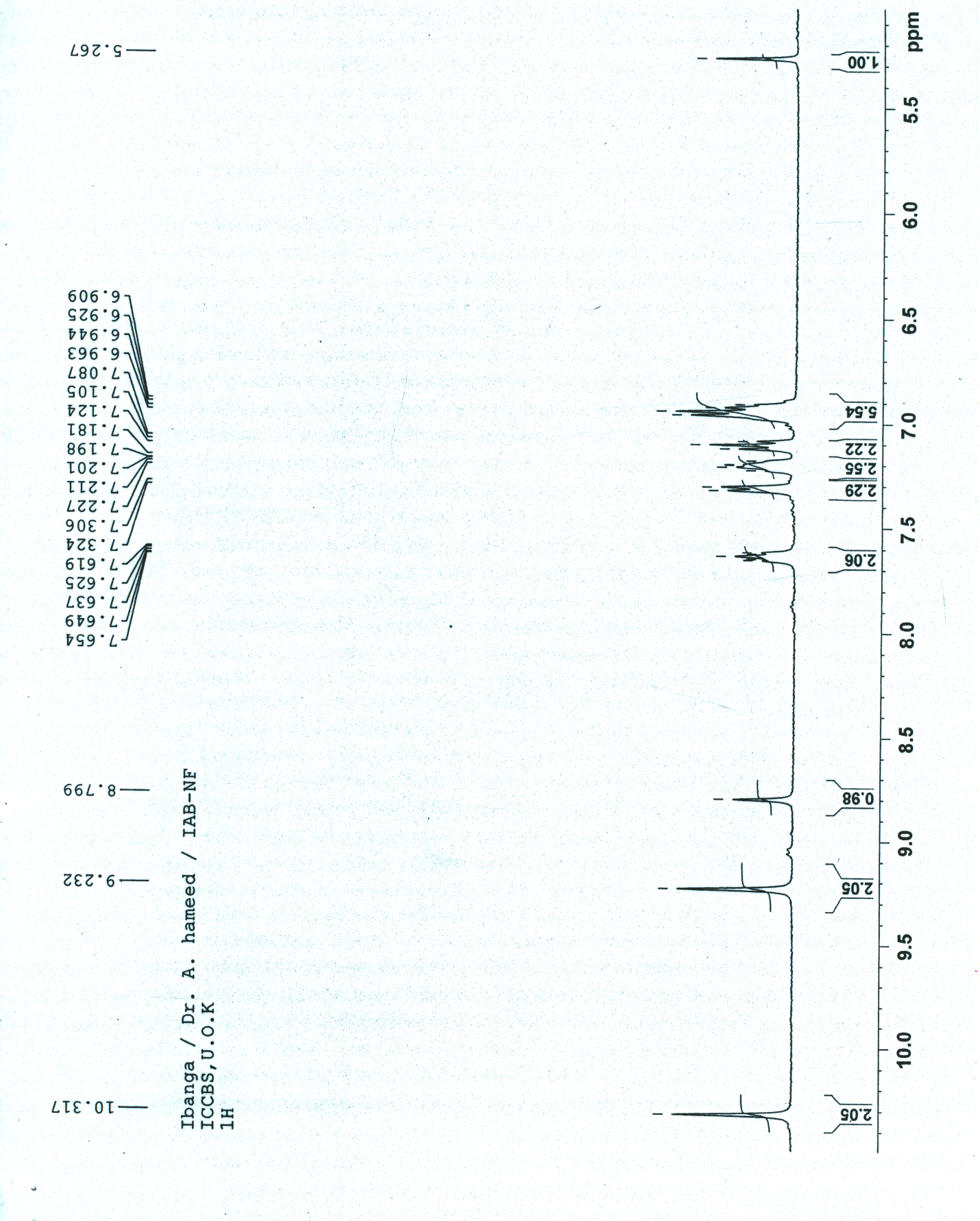
**

**
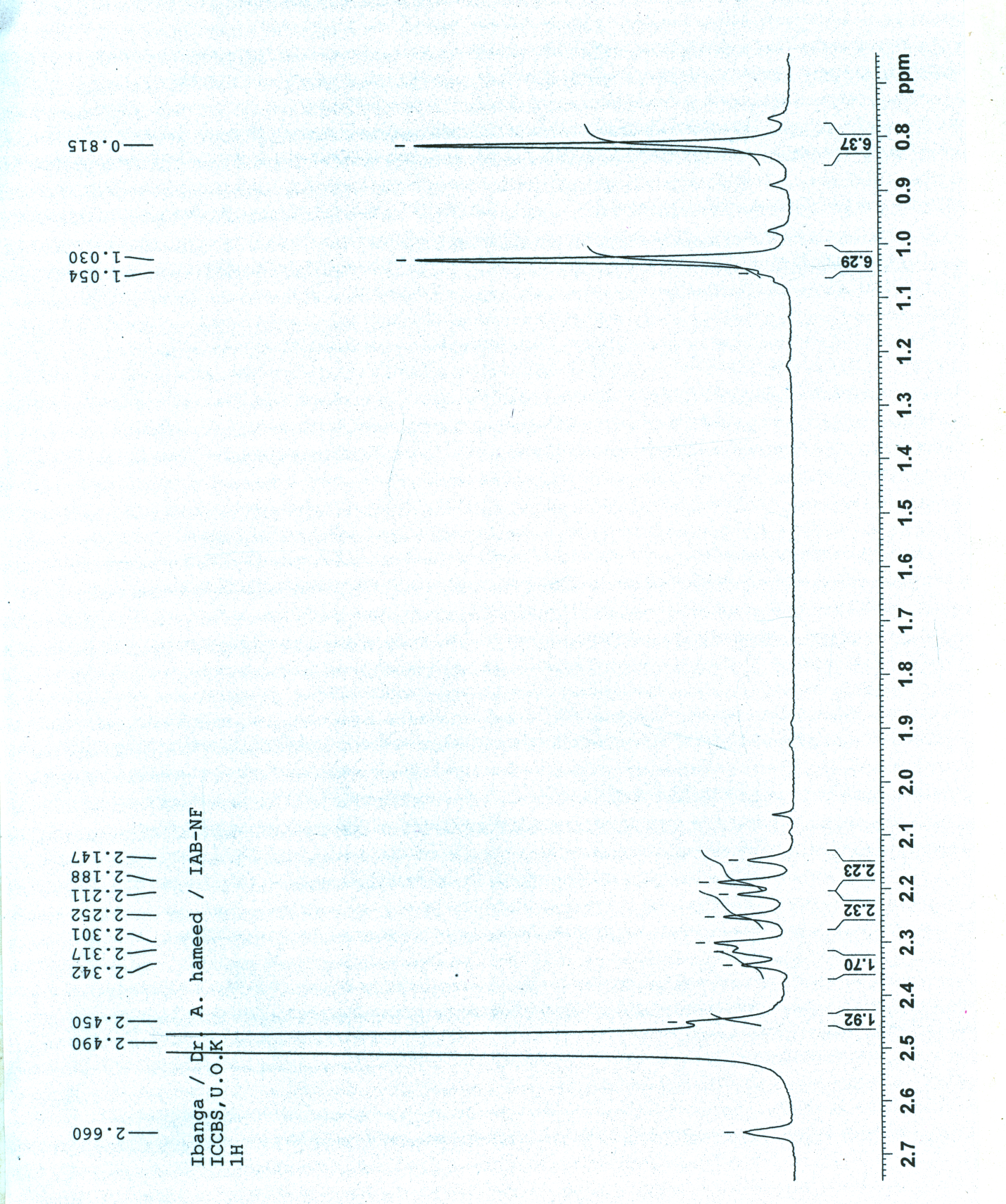

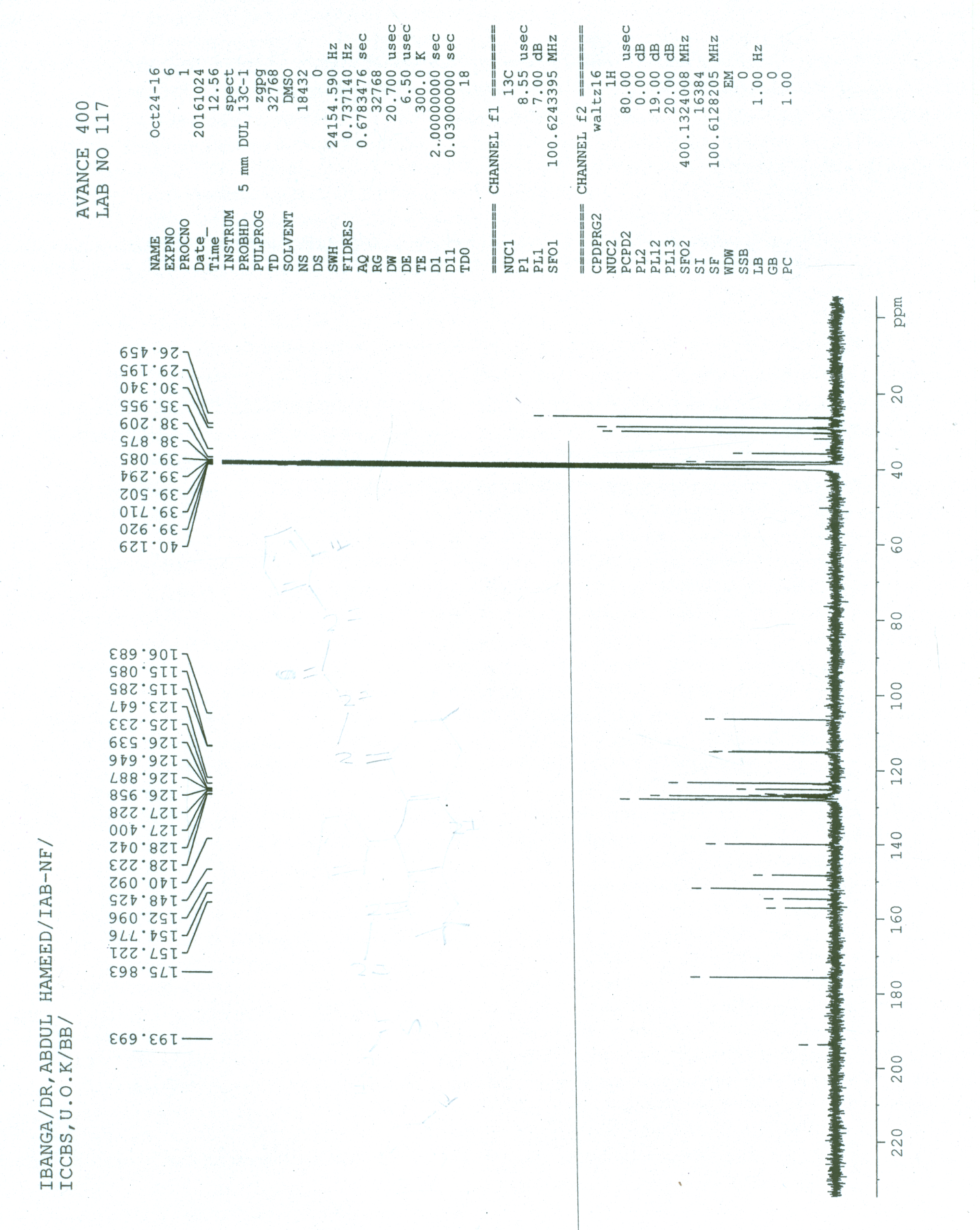

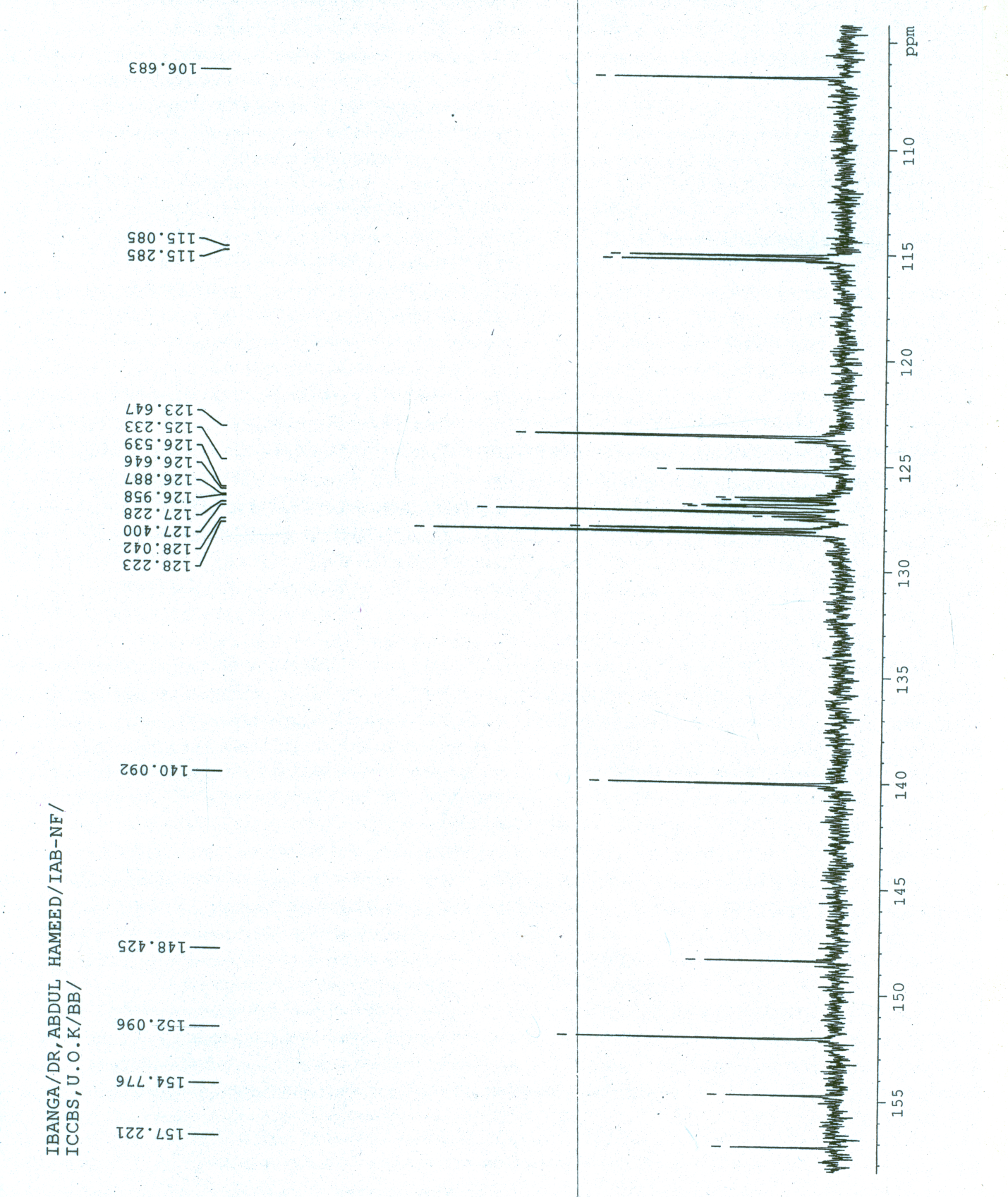

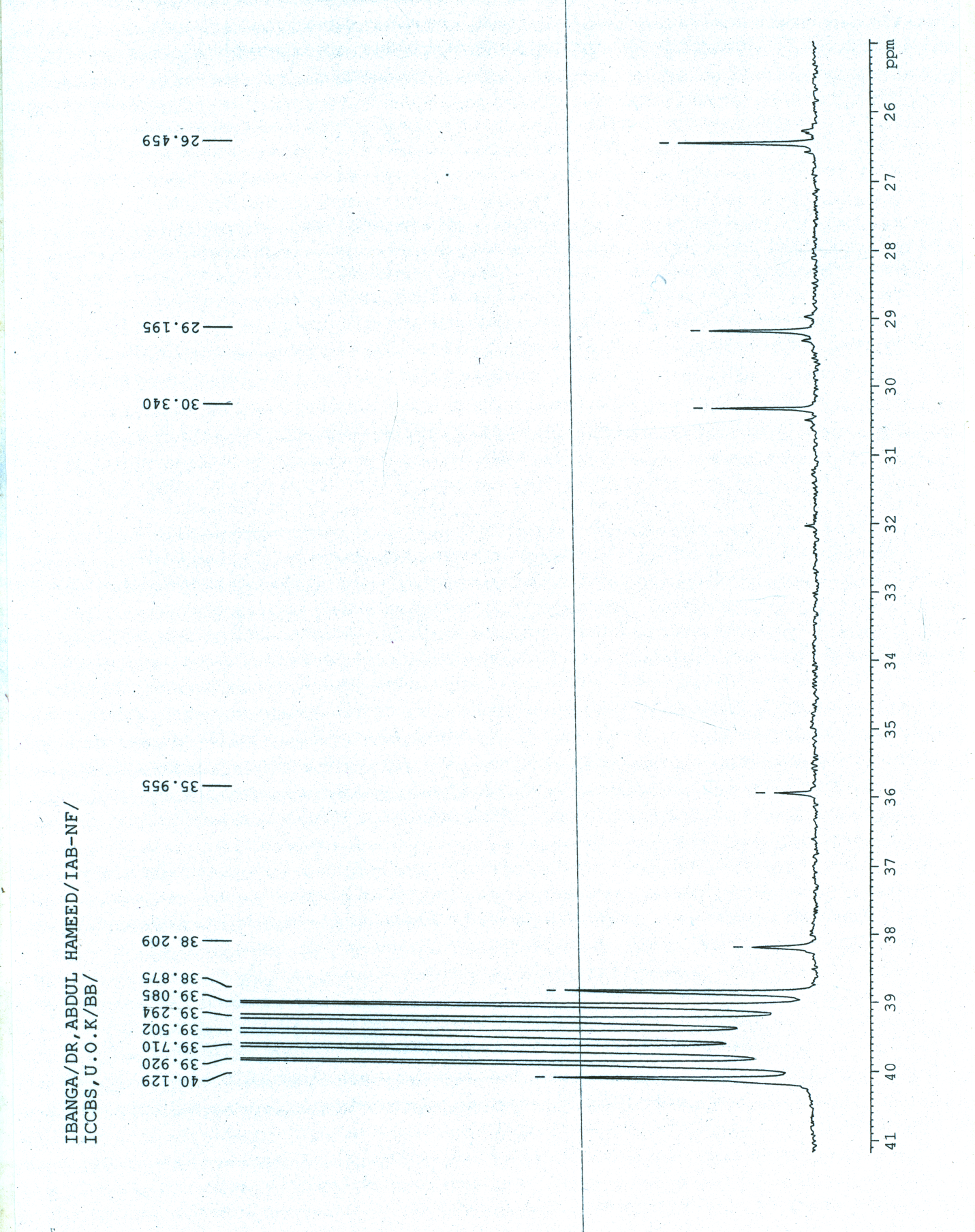
**
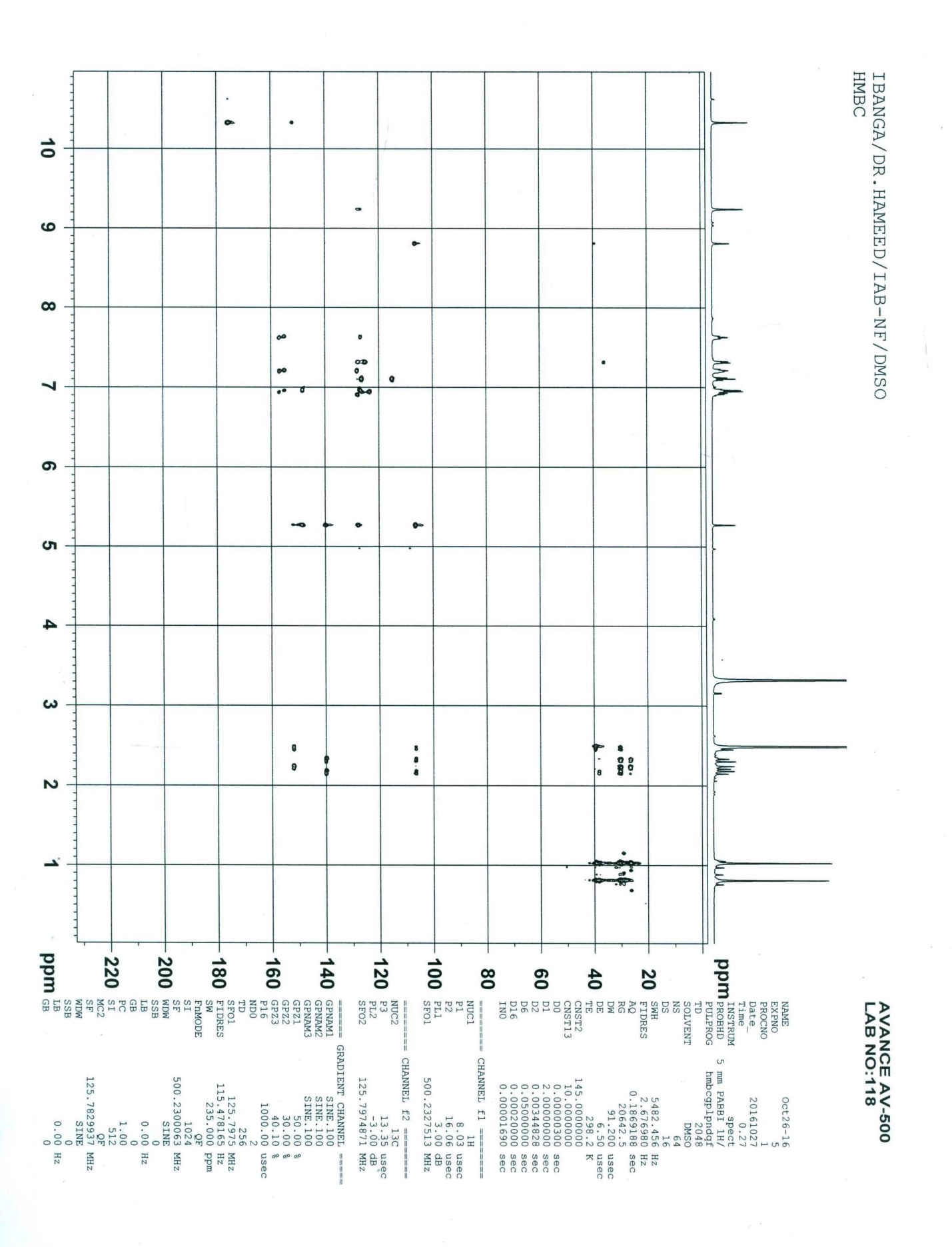

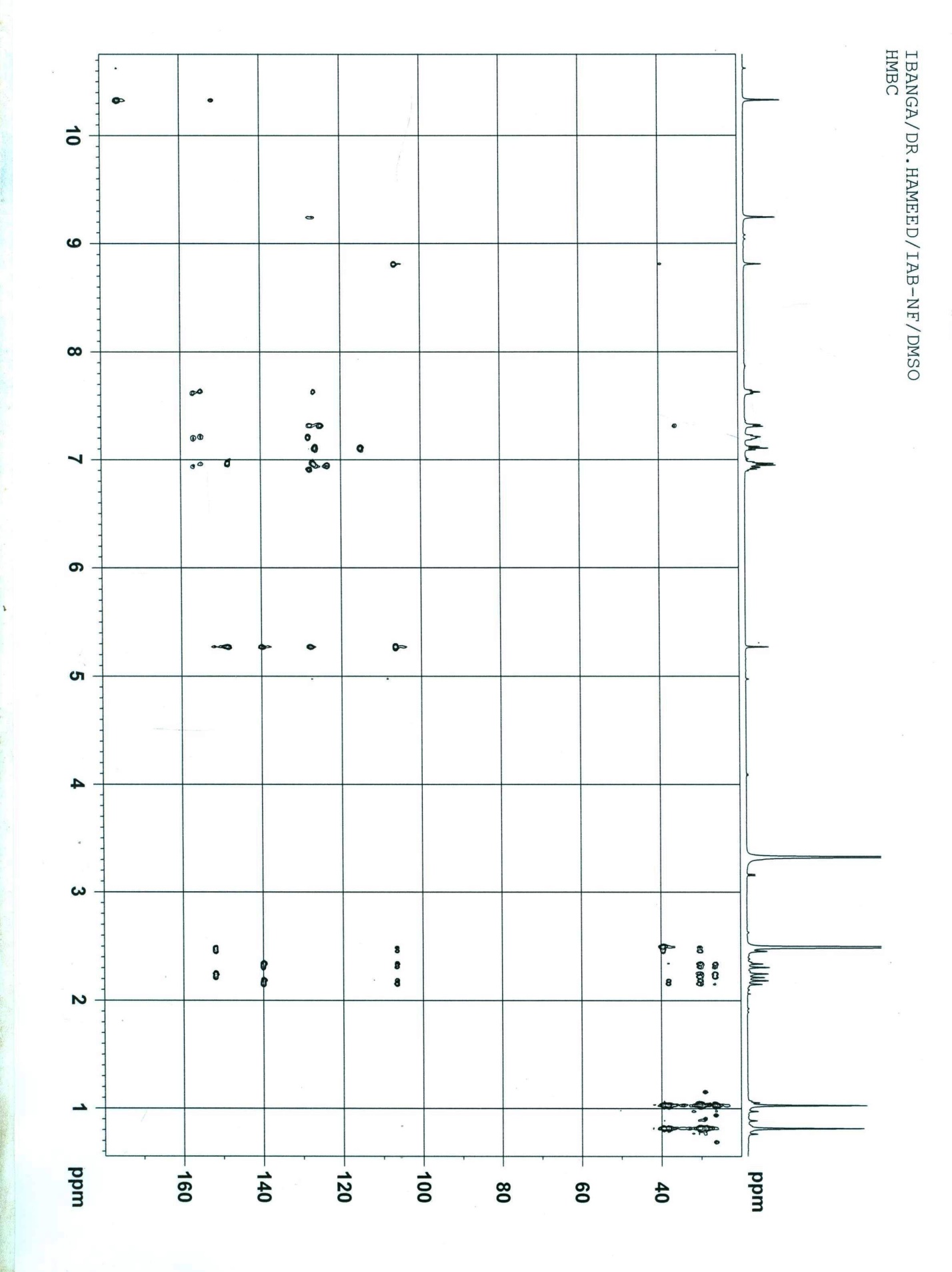

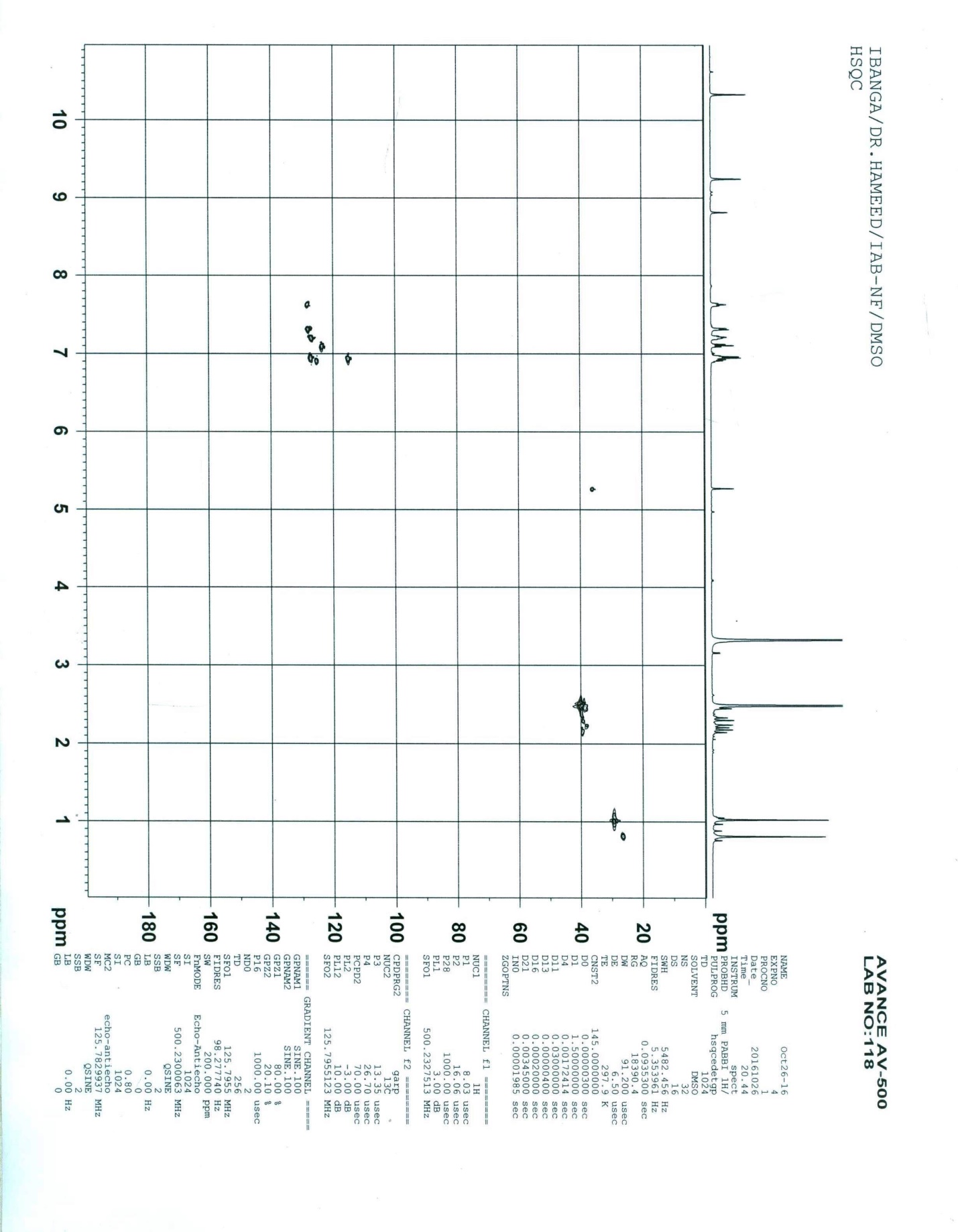

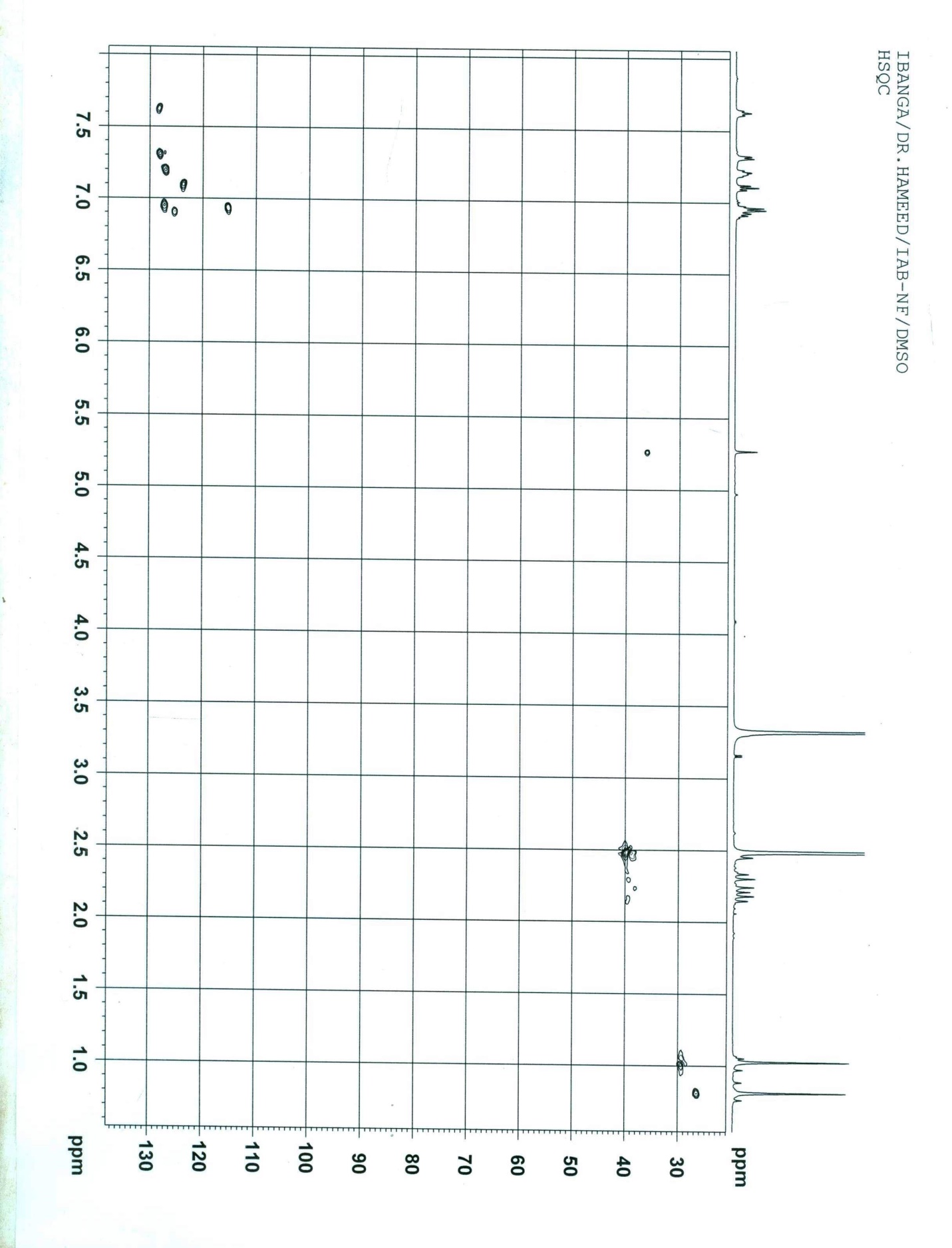
**
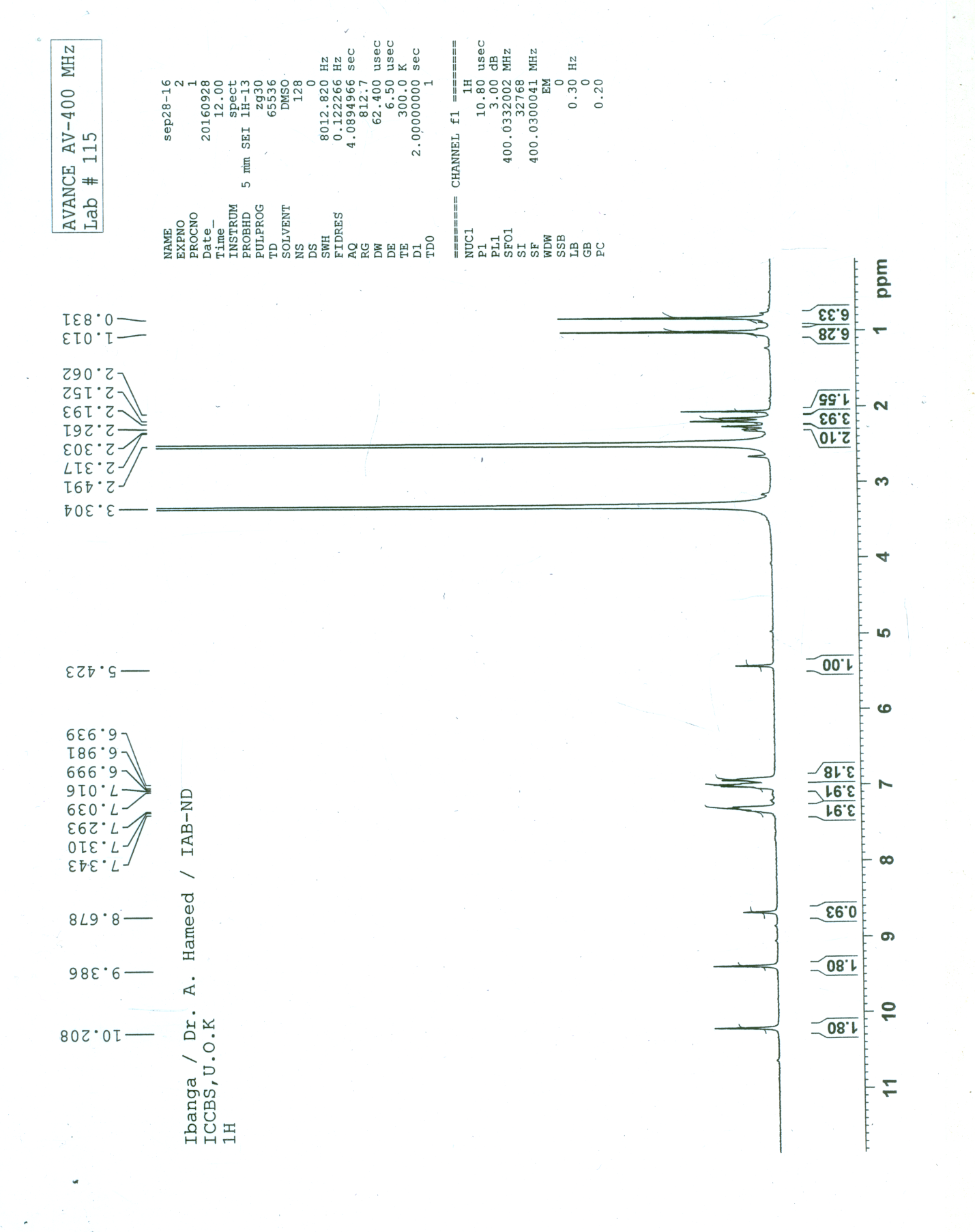

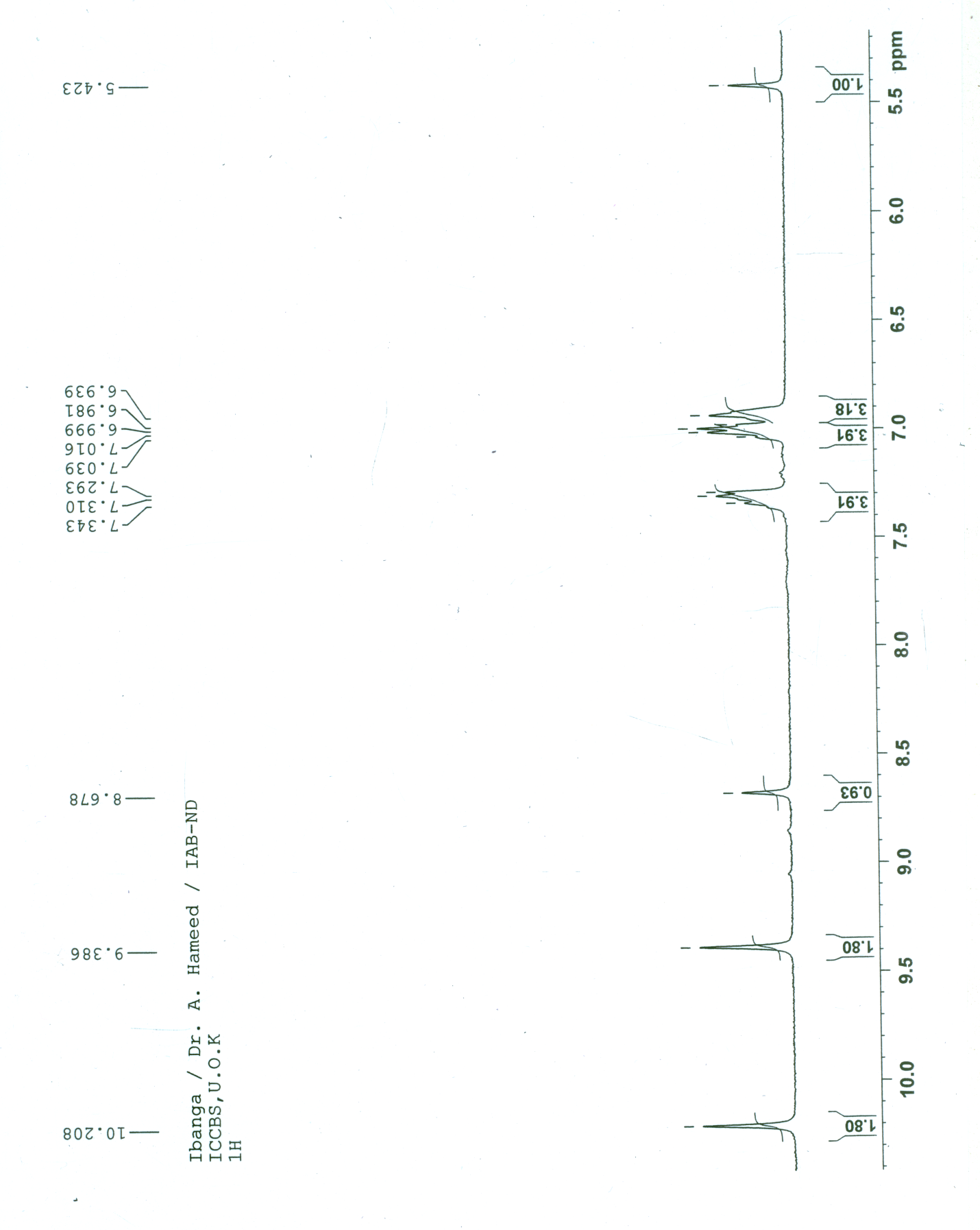

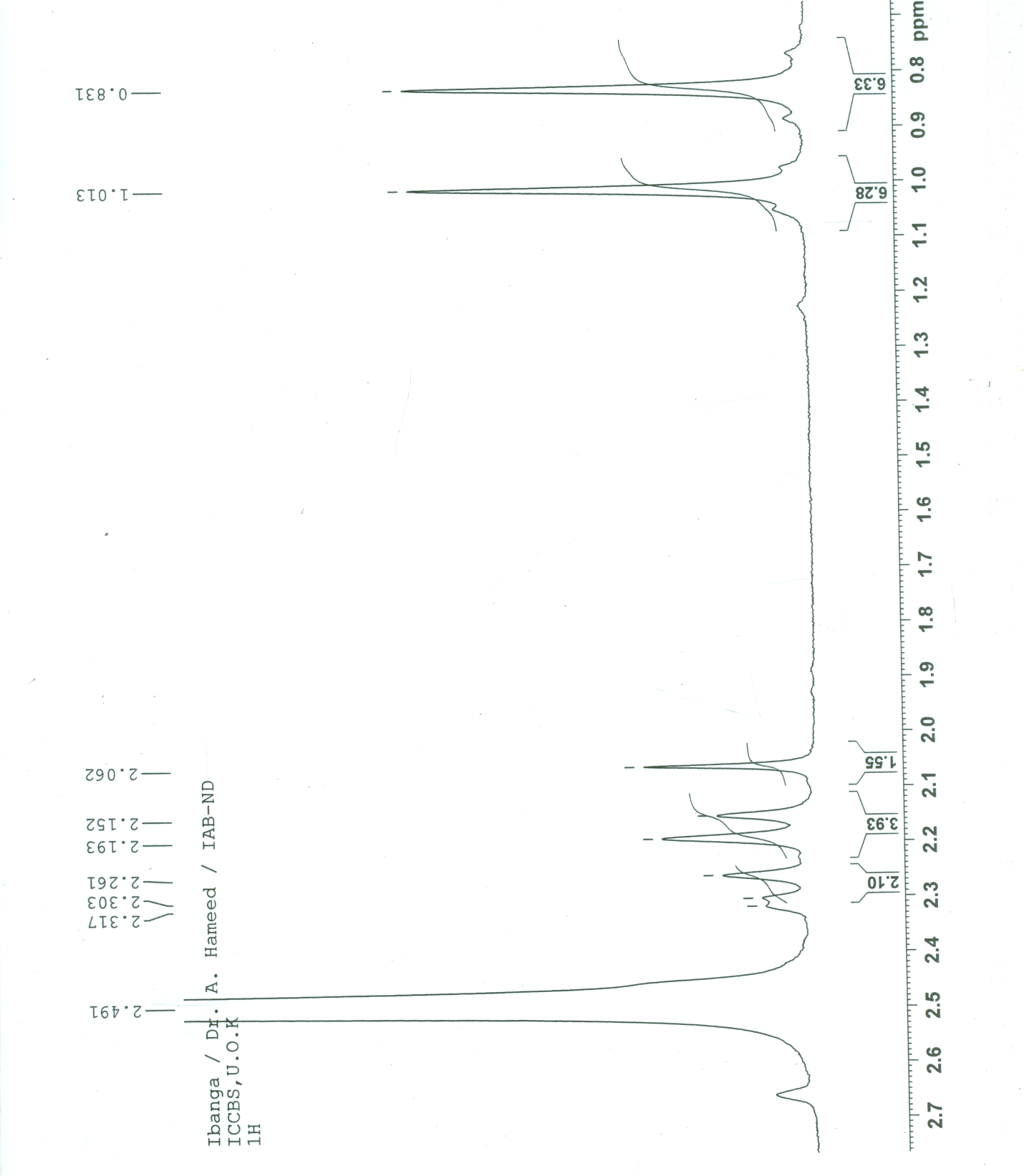
**

1. K. Ayub [khurshidayub@gmail.com](mailto:khurshidayub@gmail.com);

   A. Hameed [abdul_hameed8@hotmail.com](mailto:abdul_hameed8@hotmail.com) / [abdul.hameed@iccs.edu](mailto:abdul.hameed@iccs.edu)

   Tel.: 0092-21-99261701-2; [↑](#footnote-ref-1)
